# Activation of multiple stress responses in *Staphylococcus aureus* substantially lowers the minimal inhibitory concentration when combining two novel antibiotic drug candidates

**DOI:** 10.3389/fmicb.2023.1260120

**Published:** 2023-09-25

**Authors:** Amanda Holstad Singleton, Olaug Elisabeth Torheim Bergum, Caroline Krogh Søgaard, Lisa Marie Røst, Cecilie Elisabeth Olsen, Fredrik Heen Blindheim, Synnøve Brandt Ræder, Frithjof A. Bjørnstad, Eirik Sundby, Bård Helge Hoff, Per Bruheim, Marit Otterlei

**Affiliations:** ^1^Department of Clinical and Molecular Medicine, Norwegian University of Science and Technology (NTNU), Trondheim, Norway; ^2^Department of Biotechnology and Food Science, Norwegian University of Science and Technology (NTNU), Trondheim, Norway; ^3^Department of Chemistry, Norwegian University of Science and Technology (NTNU), Trondheim, Norway; ^4^Department of Materials Science and Engineering, Norwegian University of Science and Technology (NTNU), Trondheim, Norway

**Keywords:** antimicrobial peptide, APIM, pyrrolopyrimidine, proteomics, metabolomics, stress response, *Staphylococcus aureus*

## Abstract

The past few decades have been plagued by an increasing number of infections caused by antibiotic resistant bacteria. To mitigate the rise in untreatable infections, we need new antibiotics with novel targets and drug combinations that reduce resistance development. The novel β-clamp targeting antimicrobial peptide BTP-001 was recently shown to have a strong additive effect in combination with the halogenated pyrrolopyrimidine JK-274. In this study, the molecular basis for this effect was examined by a comprehensive proteomic and metabolomic study of the individual and combined effects on *Staphylococcus aureus*. We found that JK-274 reduced activation of several TCA cycle enzymes, likely via increasing the cellular nitric oxide stress, and BTP-001 induced oxidative stress in addition to inhibiting replication, translation, and DNA repair processes. Analysis indicated that several proteins linked to stress were only activated in the combination and not in the single treatments. These results suggest that the strong additive effect is due to the activation of multiple stress responses that can only be triggered by the combined effect of the individual mechanisms. Importantly, the combination dose required to eradicate *S. aureus* was well tolerated and did not affect cell viability of immortalized human keratinocyte cells, suggesting a species-specific response. Our findings demonstrate the potential of JK-274 and BTP-001 as antibiotic drug candidates and warrant further studies.

## 1. Introduction

The golden era of antibiotic discovery (1950–1970) led to the development of more than half the antibiotic drugs frequently used today (Davies, 2006). In contrast, few antibiotics received market approval in the last 30 years. Furthermore, many of the antibiotics that managed to gain approval operate via mechanisms similar to those already in clinical use and thus, pathogens can quickly acquire resistance (Draenert et al., 2015). The slow rate of antibiotic discovery and increasing levels of antibiotic resistance contribute to the growing difficulty in treating bacterial infections (O'Neill, 2016). To avoid a global health crisis, alternative treatment options that reduce or prevent resistance development are urgently needed.

Previously, we showed that peptides containing the proliferating cell nuclear antigen (PCNA) interacting motif APIM (APIM-peptides) inhibit DNA replication by binding to the β-clamp, the prokaryotic homolog to PCNA. At sublethal doses, APIM-peptides inhibit translesion synthesis (TLS) without blocking replication (Nedal et al., 2020). The lead antibacterial APIM-peptide, BTP-001 (β-clamp targeting peptide-001), is rapidly bactericidal in several bacterial species, including the ESKAPE pathogens, at doses that do not affect epithelization (Nepal et al., 2021). Biofilm studies demonstrated that BTP-001 eradicates existing biofilms and prevents biofilm formation in *Staphylococcus epidermidis*, and that it showed promise for use in bone cement to prevent prosthetic joint infection (Raeder et al., 2021). In addition, a recent study showed a strong additive effect of BTP-001 combined with a brominated pyrrolopyrimidine called JK-274 in *Staphylococcus aureus* (Olsen et al., 2022). The structure of JK-274 resembles that of known thymidylate monophosphate kinase (Tmk) inhibitors and has inhibitory activity against *Escherichia coli* Tmk in enzymatic assays (Blindheim et al., 2021a; Olsen et al., 2022). Combining the two drug candidates yielded an 8-fold reduction in the minimal inhibitory concentration (MIC) of JK-274 in *S. aureus* (Olsen et al., 2022).

PCNA, the eukaryotic DNA-sliding clamp, has recently been shown to also have scaffolding functions outside replication and DNA repair, e.g., in cell signaling, redox balance, and metabolism (Olaisen et al., 2015; Ohayon et al., 2019; Røst et al., 2023). As DNA sliding clamps are highly conserved, BTP-001’s mode of action (MoA) may also involve inhibiting scaffolding roles of the β-clamp outside the replisome. Identifying gene mutations in antibiotic resistant mutants is the most common method for determining the MoA of antibiotics. However, in the case of BTP-001, resistance development is very low due to the inhibition of low fidelity DNA replication by TLS polymerases (Frimodt-Møller et al., 2021; Nepal et al., 2021). Reduced resistance development confers clinical advantages but makes elucidating the MoA challenging. In this study, we investigated the individual and combined MoAs of JK-274 and BTP-001 on *S. aureus* using signalomics supplemented with metabolomics. For signalomics, we used the multiplexed kinase inhibitory bead (MIB) assay which detects the level of activated signaling proteins, many of which are involved in stress responses (Duncan et al., 2012; Petrovic et al., 2017; Røst et al., 2023). The MIB assay was optimized for bacterial cell extracts by testing several in-house compounds designed to mimic kinase inhibitors. Finally, we investigated the cytotoxicity of JK-274 and BTP-001 in combination by measuring HaCaT keratinocyte cell viability after treatment. The findings from the study present BTP-001 and JK-274 as promising antibiotic drug candidates.

## 2. Materials and methods

### 2.1. Optimization of the MIB assay for bacterial extracts

#### 2.1.1. Cell extract

MIB assay (Duncan et al., 2012; Petrovic et al., 2017) optimization was performed using pellets from *Escherichia coli* MG1655 cultivated in cation-adjusted Mueller-Hinton broth (CAMHB). Approximately 8 OD units (1 OD unit corresponds to 1 mL bacterial culture with an OD_600_ = 1.0) were sampled per pellet. Pellets were stored at −80°C until cell extraction.

To prepare the cell extract, pellets were thawed on ice and resuspended in 3× packed cell volume of lysis buffer (50% glycerol, 50 mM Tris–HCl pH 7.5, 0.1 M NaCl, 0.1 mM EDTA, 1 mM DTT, and 0.1% Triton X-100) followed by addition of OmniCleave™ Endonuclease (200 U per 100 μL; Lucigen, USA), RNAse A (1 mg/mL; Sigma Aldrich, USA), DNAse I (10 U per 100 μL; Roche, Switzerland), 1X phosphatase inhibitor cocktail 2 and 3 (Sigma-Aldrich), and 1X cOmplete™ protease inhibitor cocktail (Roche). Samples were incubated for 20 min at room temperature (RT). Lysozyme (1 mg/mL; Sigma-Aldrich) was added to the mixture and incubated for 30 min at RT. Suspensions were centrifuged (9,300 rcf, 15 min, 4°C) and the supernatant was diluted to 1 mg/mL protein.

#### 2.1.2. MIB assay

The two commercially available kinase inhibitors Purvalanol B (PurvB; Tocris, UK) and Bisindolylmaleimide-X (Bis-X; Enzo Life Sciences, USA) were tested in addition to 5 in-house investigational compounds designed to mimic kinase inhibitors referred to as L-1, L-2, L-3, L-4, and L-5 (previously SB6-060-05). The compounds L-1 and L-2 were prepared as shown in Supplementary Experimental Scheme 1 and accompanying experimental procedure. The compounds L-3, L-4, and L-5 were prepared as previously described (Bugge et al., 2016; Blindheim et al., 2021a,b). Kinase inhibitors containing carboxyl groups (PurvB, L-1, L-2, L-3, L-4, and L-5) were coupled to EAH Sepharose 4B beads (GE Healthcare, USA) and inhibitors with amino groups (Bis-X) were coupled to NHS-activated Sepharose 4 fast flow beads (GE Healthcare) according to manufacturer’s protocol.

Coupled beads (~75 μL) were added to Pierce™ spin-columns (Thermo Scientific; one column per kinase inhibitor). Bacterial extract (100 μg protein) was added to the column and incubated for 15 min at RT. The MIB assay was performed with on-column trypsinization as previously described (Petrovic et al., 2017).

#### 2.1.3. Sample preparation for analysis

After trypsinization, peptides were eluted with ammonium bicarbonate (AmBic, 0.1 M) and acetonitrile (ACN), which were collected and dried (Vacuum Concentrator plus; Eppendorf, Germany). Dried peptides were resuspended in AmBic before DTT (5 mM) was added. Samples were incubated at 55°C for 30 min on a heat block before adding iodoacetamide (IAA, 20 mM) and incubated at RT for 30 min. DTT (5 mM) was added to stop the alkylation reaction and samples were dried. Samples were resuspended in 0.1% formic acid (FA) in H_2_O. Residual detergent was removed using Pierce™ detergent removal spin columns (Thermo Scientific) according to manufacturer’s protocol. The samples were then desalted with a stage tip using two filters of C18 material. The tip was first washed with methanol (MeOH) and equilibrated with 0.1% FA-H_2_O before samples were added. The tips were washed with 0.1% FA-H_2_O and samples were eluted with 0.1% FA-ACN. The samples were dried and reconstituted in 0.1% FA-H_2_O before mass spectrometry (MS) analysis.

### 2.2. Antibiotic compounds

BTP-001 (Innovagen, Sweden and Biosynth, Netherlands) consists of the APIM-motif (in bold) connected to an arginine tail via a linker and has an acetylated N-terminus and an amidated C-terminus; Ac-MD-**RWLVK**-GILQWRKI-R11-NH_2_ (Nepal et al., 2021). Lyophilized peptide was dissolved in water and stored at 4°C. All concentrations are given as net peptide concentrations and 4 μg/mL equals approximately 1 μM. The halogenated pyrrolopyrimidine JK-274 ((*R*)-4-(4-((1-(4-bromophenyl)ethyl)amino)*-*7*H-*pyrrolo[*2,3-d*]pyrimidin-6-yl)phenol) was synthesized as previously described and 4 μg/mL is approximately equal to 10 μM (Olsen et al., 2022). JK-274 was dissolved in DMSO and stored at 4°C.

### 2.3. Bacterial strain and cultivation for signalomics and metabolomics

*Staphylococcus aureus* ATCC 29213 cultivated in CAMHB was used for signalomic and metabolomic analysis. To prepare *S. aureus* pre-cultures, glycerol stock was plated onto blood agar plates and incubated overnight at 37°C. A single colony was picked and incubated in CAMHB overnight. The pre-culture was diluted 1:100 and aliquoted into 6 individual flasks and incubated (37°C, 250 rpm) until an OD_600_ = 0.3. Next, the cultures were treated with either 0.5 μM BTP-001, 1 μM BTP-001, 5 μM JK-274, 7.5 μM JK-274 or 0.5 μM BTP-001 and 5 μM JK-274. One culture was left untreated as a control. After treatment, the cultures were incubated (37°C, 250 rpm) until sampling.

### 2.4. Signalomics and metabolomics sampling

Sampling for signalomics was conducted at 10, 25, 50 and 180 min after treatment. For the first 3 timepoints, 10 mL culture was sampled from 3 independent biological replicas (BRs). At 180 min, 5 mL were sampled from 4 BRs. Samples were centrifuged (4,500 rcf, 10 min, 4°C) and supernatant discarded. The pellet was frozen in N_2_ (*l*) and stored at −80°C until further processing.

Samples for metabolic profiling were harvested from 4 BRs 180 min after treatment. The sampling procedure was adapted for *S. aureus* from a previously described protocol developed for *E.coli* (Thorfinnsdottir et al., 2023). Briefly, biomass equivalent to 10 OD units was sampled and filtered through two stacked polyvinylidene fluoride filters with a pore size of 0.65 μm (Durapore^®^, DVPP04700; Millipore, USA) exposed to a vacuum pressure of 400 mbar below the ambient pressure. Filtered biomass was rinsed with Milli-Q H_2_O (10 mL, 37°C), quenched in 10 mL ice-cold MeOH:ACN:H_2_O (20:30:50), frozen in N_2_ (*l*) and kept at −80°C until further processing.

### 2.5. Signalomics sample preparation and analysis

*Staphylococcus aureus* cell extract was prepared as described for *E. coli* in MIB optimization, except lysostaphin (1 μg/mL) was used instead of lysozyme. Cell extract (100 μL; 1 mg/mL protein) was added to an equal mix of Sepharose beads coupled to the kinase inhibitors PurvB, L-1 and L-3. The MIB assay and sample preparation for MS was conducted as described for MIB optimization.

For both *E. coli* (MIB optimization) and *S. aureus* samples, liquid chromatography (LC)-MS/MS analysis was used for protein identification and MS data were processed using MaxQuant v 2.1.3.0 for label free quantification (LFQ) of proteins (Cox et al., 2014). The following search parameters were used: the digestion enzyme was specified as trypsin with a maximum of 2 missing cleavages, variable modifications were set to oxidation (M), acetylation of protein N-terminal, and deamination (NQ), and fixed modifications were set to carbamidomethyl (C). LFQ min. ratio count was set to 1. Samples were queried against the imported *E. coli* (strain K12, proteome ID: UP000000625, downloaded February 18, 2021) or *S. aureus* (strain NCTC 8325/PS 47, proteome ID: UP000008816, downloaded on February 09, 2022) reference proteome from UniProt and Andromeda, MaxQuant’s internal contaminants database. The false discovery rate (FDR) for protein and peptide identification was set to 1%. Only unique peptides were used for definite protein group identification. The area under the peak curve was integrated to obtain peak abundances. The total abundance of all peptides identified for each protein during each run was used to normalize the abundance in every protein group using the LFQ algorithm with minimum peptides ≥1.

LFQ values were log-transformed with base 2 before subtracting the control for each timepoint, respectively, to yield the change relative to the control for each treatment group. The values were then tested using a two-sided Wilcoxon signed rank test in R v 4.2.1 to check the directionality of change for each treatment group compared to the control. To reduce the number of hits, LFQ values were also analyzed in R using the DEP package v 1.18.0 from Bioconductor v 3.16 (Zhang et al., 2018). Each timepoint was analyzed separately. Data were normalized using variance stabilizing transformation. Proteins with a missing value in ≥2 BRs for more than 1 sample were filtered out, and remaining missing values were imputed by the k-nearest neighbor (kNN) technique. Differentially pulled down proteins were defined as having either an increased or decreased pulldown compared to the control in all BRs (based on the Wilcoxon signed rank test) and having a *p*-value ≤0.1. The STRING v 11.5 database was used to create a protein–protein physical interaction network of differentially pulled down proteins with the highest confidence (interaction score > 0.9) (Szklarczyk et al., 2023). The PANTHER v 17.0 classification system was used to run a gene ontology enrichment analysis of biological processes (Mi et al., 2013).

The LC–MS/MS signalomics data have been deposited in the ProteomeXchange Consortium via the PRIDE (Perez-Riverol et al., 2021) partner repository with the dataset identifier PXD043757.

### 2.6. Metabolomics sample preparation and analysis

Intracellular metabolites were extracted by an extended extraction protocol based on Thorfinnsdottir et al. (2023). Briefly, samples were cycled between −20°C EtOH and N_2_ (*l*) in three consecutive freeze–thaw cycles before filters were removed and remaining cell debris was pelleted (4,500 rcf, 10 min, −9°C). Supernatants were lyophilized, reconstituted in 500 μL cold MQ-H2O and cleared by spin-filteration with a 10 kDa molecular cutoff (20,817 rcf, 10 min, 0°C).

Phosphorylated metabolites, organic acids, and amino acids were absolutely quantified by capillary ion chromatography (capIC-) and LC–MS/MS as previously described (Stafsnes et al., 2018; Røst et al., 2020).

Downstream data processing and absolute quantification was performed as previously described applying the TargetLynx application manager of MassLynx 4.1 (Waters, USA). Absolute concentrations were interpolated from calibration curves prepared from appropriate dilutions of analytical grade standards (Sigma-Aldrich). The response factor of the corresponding U^13^C-isotopologue of each compound was used to correct the standard and sample extract response factors. Extract concentrations were normalized within replicas and to the total metabolite abundance for all treatments. T-tests were conducted in MetaboAnalyst to identify metabolites with a significant change compared to the control (Pang et al., 2020).

### 2.7. FtsZ live cell microscopy

Exponentially growing *S. aureus* were washed 3× with PBS and the pellets were resuspended in PBS containing 2 μg/mL BioTracker Bacterial FtsZ Cell Dye (Sigma-Aldrich; SCT090, excitation: 488 nm, emission: 510 nm) (Ferrer-González et al., 2019) and incubated at RT for 30 min. The samples were washed 2× with PBS and the pellets were resuspended in PBS (volume based on cell density). Then, 8 μL were added to an agarose pad to immobilize the bacterial cells before examination in a LSM 510 Meta laser scanning microscope (Zeiss, Germany) equipped with a plan-apochromat 63×/1.4 oil immersion objective.

### 2.8. Immunoprecipitation and His-tag purification assays

#### 2.8.1. Cell extracts of overexpressed APIM-EYFP and His-tagged β-clamp

Overnight cultures of *E. coli* BL21 cells containing plasmids for expression of EYFP-tagged APIM-peptide or His-tagged β-clamp (pET28/pET16b, respectively) were diluted 1:100 and grown in LB to OD_600_ = 0.3–0.4 before protein expression was induced with isopropyl β- d-1-thiogalactopyranoside (IPTG, 300 nM). As controls, BL21 cells expressing only EYFP and only His-tag were used. Pellets from bacterial culture from each sample were collected 2 h after IPTG induction by centrifugation (3,220 rcf, 10 min). APIM-EYFP expressing cells were crosslinked by resuspending the pellets in PBS containing 1% formaldehyde and incubating for 20 min at RT. Crosslinking was quenched with 2.2 mM glycine for 5 min before pellets were washed 3× with PBS. Cell extracts were prepared as described for MIB optimization with the addition of Benzonase^®^ Nuclease (250 units, Sigma-Aldrich) and sonication (3 × 30 s) instead of lysozyme.

#### 2.8.2. IP with EYFP-tagged APIM-peptide

Dynabeads^®^ Protein A (4.5 mg, Invitrogen, USA) were washed with conjugation buffer (20 mM NaP and 0.15 M NaCl pH 7.9) and incubated in conjugation buffer with 35 μg antibody of α-GFP (Abcam, UK; ab290) for 1 h. Beads were crosslinked with BS^3^ (bis(sulfosuccinimidyl)suberate, Thermo Scientific) according to the manufacturer’s manual. The beads were washed 3× with IP buffer I (20 mM HEPES, 1.5 mM MgCl_2_, 200 mM KCl, 0.2 mM EGTA, 20% (v/v) glycerol and 0.5% NP-40) and finally resuspended in IP buffer II (20 mM HEPES, 1.5 mM MgCl_2_, 200 mM KCl, 0.2 mM EGTA, 10% (v/v) glycerol). Each IP reaction, containing 0.5 mg cell extract, 0.6 mg conjugated beads and IP buffer II, was incubated at 4°C overnight on rotation. Further, the IP reactions were washed once with IP buffer II and 3× with washing buffer (10 mM Tris–HCl pH 7.5 and 600 mM NaCl). The pelleted beads were then prepared for MS analysis.

#### 2.8.3. His-tag purification assay with His-tagged β-clamp

TALON^®^ Metal Affinity Resins (Clontech Laboratories, Inc., USA) were used to pull down the His-tagged β-clamp proteins and its interaction partners. Each sample required 0.4 mL resin which was equilibrated by washing the resin 3× in basis buffer (50 mM NaP pH 8, 300 mM NaCl, 0.01% tween, 10 mM β-mercaptoethanol) followed by centrifugation (700 rcf, 5 min). The cell extracts were added to the resin and incubated for 1 h at 4°C followed by another centrifugation step. The resin was resuspended in wash buffer (basis buffer containing 10 mM imidazole) which was added to a Poly-Prep^®^ Chromatography Column (Bio-Rad, USA) where the resin was washed three additional times with washing buffer (gravity flow). After the last wash, the tip was closed and the resin resuspended with basis buffer. This was transferred to a tube and centrifuged (700 rcf, 5 min). The pelleted resin was then prepared for MS analysis.

#### 2.8.4. Preparation of samples for MS analysis

The beads from the EYFP IP assay and the resin from the His-tag purification assay were digested with trypsin and prepared for MS analysis by desalting with a C18 stage tip. First, 50 mM AmBic and 200 mM DTT were added to the beads/TALON resin, followed by incubation at 55°C for 30 min. After cooling to RT, 200 mM IAA was added and the samples were incubated in the dark for 30 min. Trypsin (1.5 μg) was added and the samples were shaken overnight at 37°C. The following day, the beads were removed and washed with AmBic. The wash, together with the rest of the sample, was dried before reconstituted in 0.1% FA-H_2_O. The samples were then desalted as described for MIB optimization before MS analysis.

### 2.9. Reactive oxygen species (ROS) assay

ROS were measured using the cell-permeable probe 2′,7′-dichlorofluorescein diacetate (DCFH-DA; Sigma-Aldrich). Briefly, the overnight culture was centrifuged (3,220 rcf, 10 min) and resuspended in PBS to OD_600_ = 0.3. The resuspended culture was incubated with DCFH-DA (5 μM) for 30 min (25°C, 250 rpm). A 96-well plate was prepared with the different concentrations of BTP-001 and positive control wells of H_2_O_2_ (20 mM), while some wells were left as untreated control wells. The culture with loaded probe was added to the plate to a total volume of 200 μL in each well. The plate was incubated for 30 min (37°C, 400 rpm) before measuring fluorescence (excitation at 485 nm, emission at 520 nm).

### 2.10. Viability assay

Cytotoxicity of JK-274 on human cells was tested on HaCaT (spontaneously transformed aneuploid immortal keratinocyte cell line) using the PrestoBlue™ viability assay (Invitrogen) according to the guidelines provided by the manufacturer. Briefly, 3,000 cells/well were seeded in 96-well plates. After adhesion (minimum 4 h), the cells were treated with JK-274 (2.5–20 μM) and BTP-001 (2 μM) or left untreated and incubated (37°C, 5% CO_2_, humidified atmosphere). The media was either left unchanged or exchanged for fresh media 4 h after treatment. Viability was measured 24 h after treatment by adding PrestoBlue™ to a final concentration of 1× and incubating for 2 h at 37°C before reading the fluorescence (excitation at 544 nm, emission at 590 nm).

To measure viability of *S. aureus* after 4 h of treatment with JK-274 and BTP-001, 3 × 10^8^ CFU/mL were added to a 96-well plate and treated with either JK-274 (2.5 μM) and BTP-001 (2 μM), isopropanol (100%), or left untreated, and incubated for 4 h (37°C). Viability was measured as described above. CFU/mL was also determined after 4 h.

## 3. Results and discussion

In this study, we first compare a group of kinase inhibitors to determine the optimal set for signalomics analysis of bacterial cell extracts using the MIB assay. Next, we present a general overview of the study design and main characteristics shared across all treatment groups before giving an in-depth analysis of the specific effects seen in *S. aureus* after exposure to JK-274 and BTP-001 separately and in combination. Finally, the cytotoxicity on mammalians cells was assessed.

### 3.1. Optimization of the MIB assay for prokaryotes

**Figure 1:**
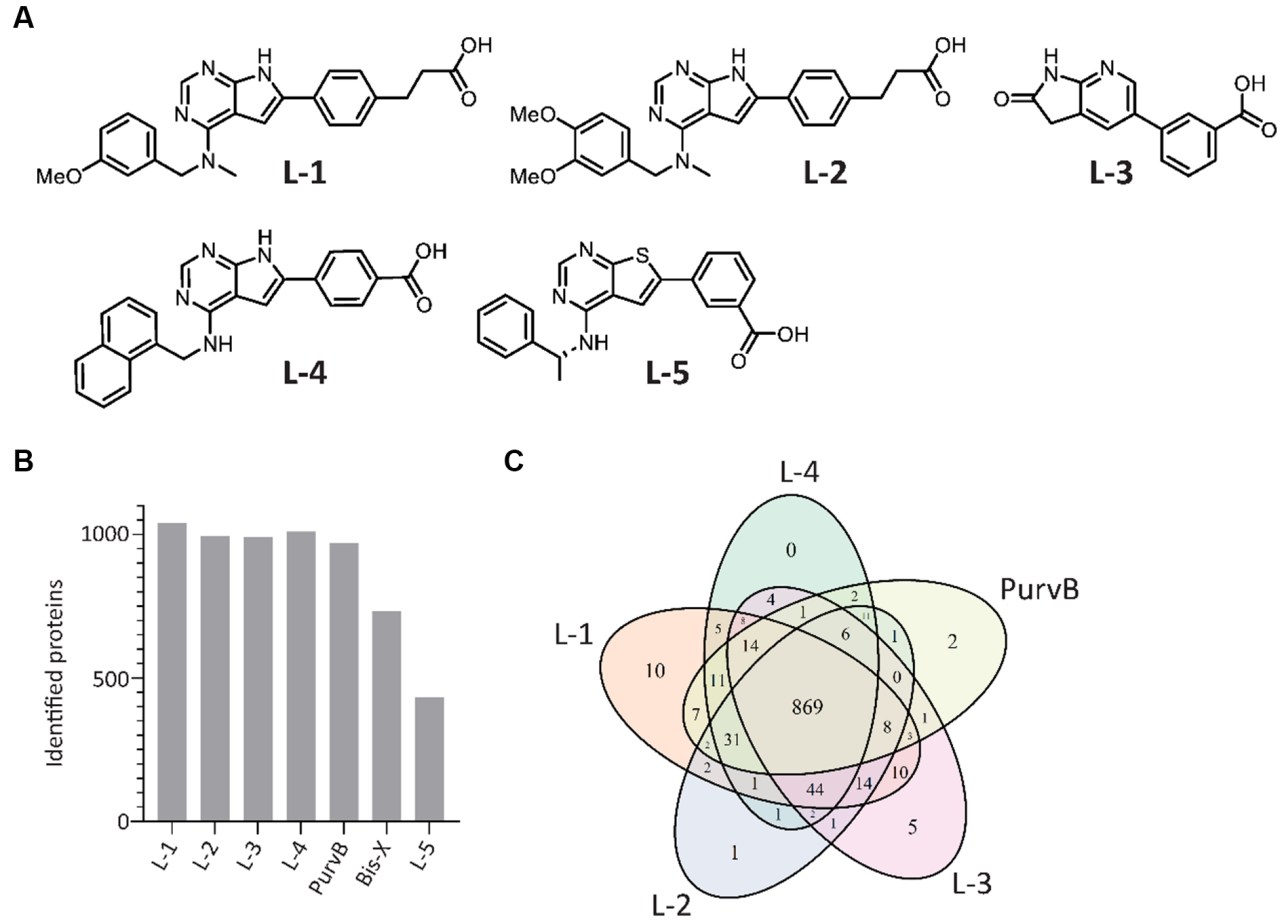
In-house and commercial kinase inhibitors exhibit overlap in protein pulldown. **(A)** Chemical structures of the 5 in-house kinase inhibitors. **(B)** Number of *E. coli* proteins identified using the 7 tested kinase inhibitors during MIB assay optimization. Proteins were detected by LC–MS/MS and quantified using MaxQuant. **(C)** Venn diagram of the 5 kinase inhibitors with the highest number of proteins pulled down.

The proteome contains thousands of proteins with diverse functions, some of which may not be relevant to determining antibiotic MoA. In addition, signal transduction in response to stimuli such as antibiotics is a rapid and transient event that occurs much faster than protein synthesis and degradation. Selecting for the signalome reduces the complexity of the results, making it easier to identify key proteins and signaling pathways, and allows for the detection of dynamic changes caused by post-translation modifications that alter protein activity. The MIB assay has successfully been used to study the signalome in both eukaryotic and prokaryotic cells by pulling down activated signaling proteins, such as kinases, ATP/GTP-binding proteins, and proteins in complex with the latter two (Petrovic et al., 2017; Søgaard et al., 2019; Sumabe et al., 2021; Røst et al., 2023). Increased protein pulldown upon treatment suggests increased activation while reduced pulldown indicates inhibition, destabilization, or reduced activation of the protein. The kinase inhibitors L-5 (in-house), PurB, and Bis-X were used in previous studies, but with a greater ability to pull down proteins in eukaryotes than prokaryotes. To optimize the MIB assay for prokaryotes, we compared the previously used kinase inhibitors to 4 in-house compounds designed to mimic kinase inhibitors (from here on referred to as kinase inhibitors) for their ability to pull down proteins from the model organism *E. coli* MG1655. The chemical structures for the in-house kinase inhibitors are shown in Figure 1A.

In total, the kinase inhibitors pulled down 1,077 unique proteins, with L-1, L-2, L-3, L-4, and PurvB pulling down the highest number of proteins (Figure 1B). The 5 kinase inhibitors with the highest pulldown had 869 proteins in common (Figure 1C). The two kinase inhibitors not included in the Venn diagram, Bis-X and L-5, did not pull down any unique proteins. The best combination was determined to be a mixture of L-1, L-3 and PurvB, which included 1,075 proteins, accounting for 24% of the *E. coli* MG1655 proteome. This set of kinase inhibitors yielded even better pulldown in *S. aureus* as they pulled down 1,568 proteins, covering ~50% of the total proteome (raw data uploaded in PRIDE). The results indicate that the MIB assay can be used for multiple bacterial species but that the proteome coverage may vary.

To check if the kinase inhibitor concentrations used in the MIB assay were sufficient to prevent abundant proteins from saturating the kinase inhibitors and outcompeting scarce proteins, we increased the amount of kinase inhibitor added to the MIB column. The number of detected proteins remained the same; thus, the ratio of pulled down proteins likely reflects the ratio found in the cell.

### 3.2. JK-274 and BTP-001 reduce the growth of exponentially growing *Staphylococcus aureus* more in combination than as single agents

**Figure 2:**
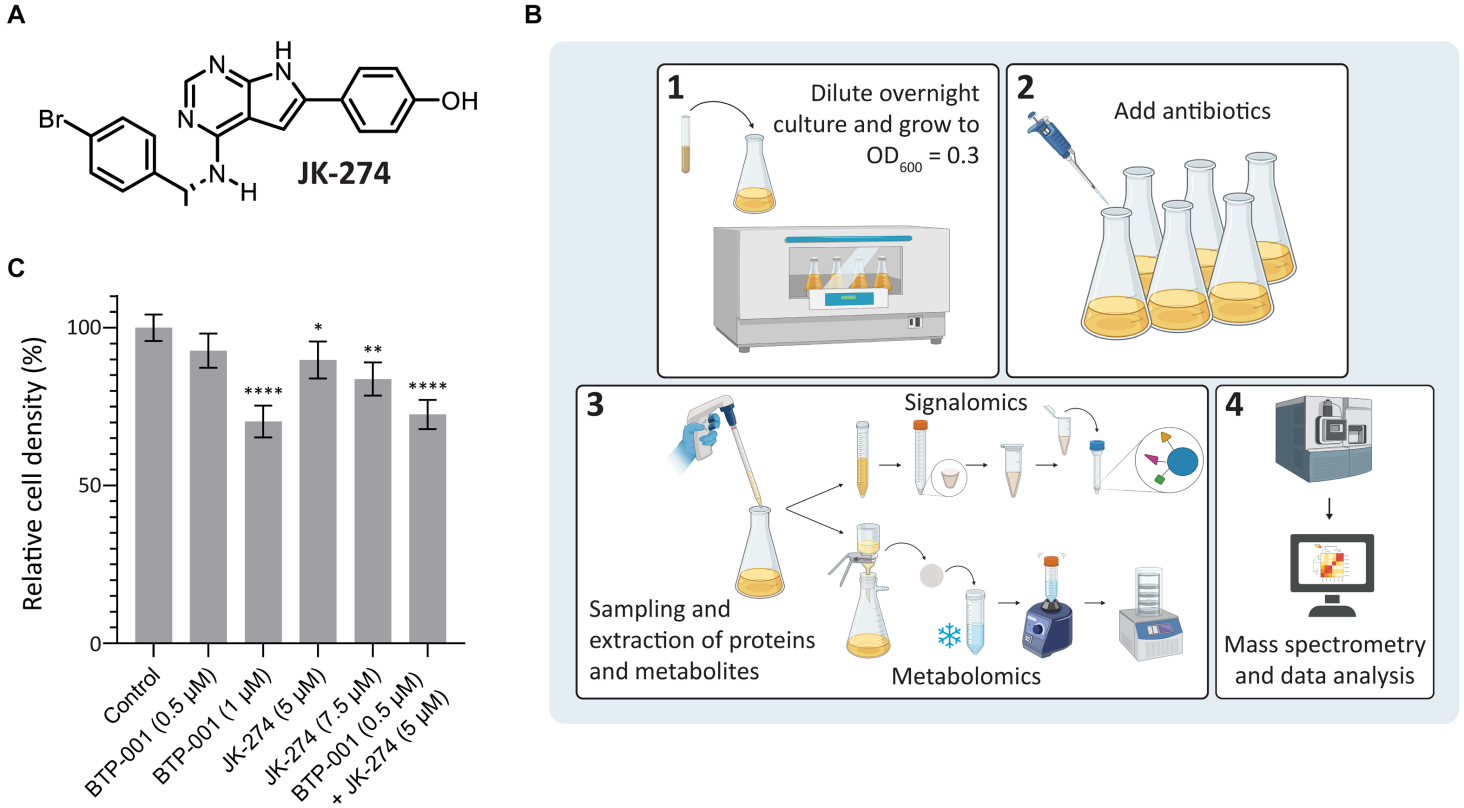
Study design and cell densities after treatment. **(A)** Chemical structure of JK-274. **(B)** Overview of the signalomics and metabolomic workflow. First (1), the overnight culture is diluted and grown to OD_600_ = 0.3 before (2) antibiotic treatment is added. Then, (3) samples are collected for signalomics and metabolomics before subsequent extraction. Signalomics samples are pelleted to make cell extract for the MIB assay. Metabolite samples are freeze-thawed with vigorous vortexing to extract metabolites before lyophilization. Finally, (4) mass spectrometry and data analysis are conducted to identify proteins and metabolites. **(C)** Cell density of *S. aureus* 3 h after treatment with JK-274 (5 and 7.5 μM) and BTP-001 (0.5 and 1 μM), separately and in combination (only the lower concentrations) given relative to an untreated control. Mean ± SD, *n* = 4. One-way ANOVA (control vs. treatment): *p < 0.05, **p < 0.01, ****p < 0.0001.

A putative Tmk inhibitor called JK-274 (Figure 2A) was previously found to have a low MIC (20 μM, 8 μg/mL) toward *S. aureus*. When combined with a ½ MIC BTP-001 (2 μM, 8 μg/mL), the MIC for JK-274 was reduced 8-fold (Olsen et al., 2022). In this study, we wanted to investigate the molecular basis of the individual and combined effects of JK-274 and BTP-001 by using signalomics supplemented with metabolomics. Our goal was to treat exponentially growing *S. aureus* with doses that induce stress without substantial lethality to explore the specific effects of JK-274 and BTP-001 while avoiding non-specific changes caused by extensive cell death. Sub-MIC doses also guarantee sufficient biomass for signalomic and metabolomic sampling. To ensure sampling during logarithmic growth, the *S. aureus* cultures received sub-MIC treatment at OD_600_ = 0.3 (experimental scheme shown in Figure 2B). Growth curves from preliminary studies were used to determine the doses to be used in combination (Supplementary Figure S1). The untreated control reached an OD_600_ = 1.6 in 3 h (Figure 2C). Single treatments with 5 μM JK-274 (1/4 MIC) and 0.5 μM BTP-001 (1/8 MIC) almost reached the same cell density as the untreated control; however, when combined, the two doses yielded a reduction in cell density (Figure 2C). To compare the combination treatment to single treatments with approximately the same cell density, we also treated *S. aureus* with 1 μM BTP-001 and 7.5 μM JK-274.

### 3.3. An overview of the systemic response to JK-274 and BTP-001

**Figure 3:**
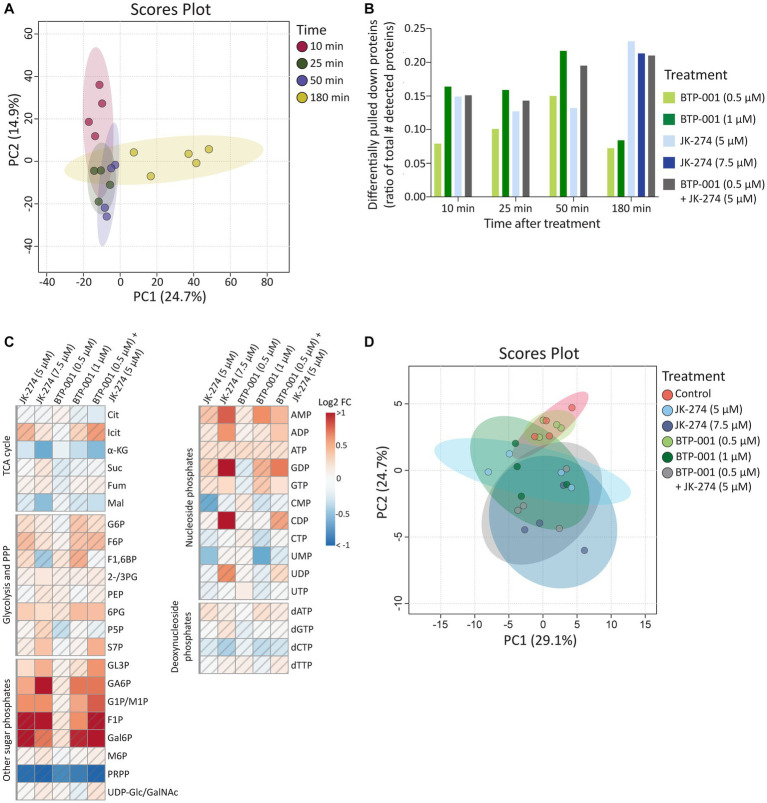
The systemic changes caused by JK-274 and BTP-001 as single agents and in combination. **(A)** Scores plot from PCA of the log_2_ fold change compared to the control of protein pulldown after treatment with JK-274 (0.5 and 1 μM) and BTP-001 (5 and 7.5 μM), separately and in combination (only the lower concentrations) at 10, 25, 50, and 180 min after treatment. Auto-scaling was applied; 95% confidence regions indicated. **(B)** Differentially pulled down (DPD) proteins after JK-274 and BTP-001 treatment. DPD proteins are shown as a ratio of the total number of detected proteins at each time point. DPD proteins are significant according to the Wilcoxon signed rank test and have a value of *p* ≤0.1 using moderated T-statistics. **(C)** A heatmap displaying the log_2_ fold change (FC) for a select group of intracellular metabolites 180 min post-treatment given relative to an untreated control. A hatched field indicates a value of *p* > 0.1 based on a *t*-test between the treatment and control samples. Quantified metabolites are listed in Supplementary Table S2 and abbreviations are listed in Supplementary Table S3. **(D)** Scores plot from PCA of the intracellular metabolite pool concentrations. Auto-scaling was applied; 95% confidence regions indicated.

As mentioned above, 1,568 proteins were pulled down from *S. aureus* using the MIB assay (Supplementary Table S1). Principal component analysis (PCA) revealed that samples from the same timepoint, independent of treatment, cluster together (Figure 3A); therefore, we only compared samples taken at the same timepoint. Many proteins exhibited differential pulldown (DPD) after treatment compared to the control, with BTP-001 yielding the most DPD proteins at 10, 25, and 50 min post-treatment while JK-274 and the combination resulted in the most DPD proteins at 180 min (Figure 3B); thus, indicating that their effects on the signalome are temporally separated. For metabolomics, the intracellular concentrations of selected metabolites were quantified at 180 min post-treatment (Supplementary Table S2), which may have been too late to detect major changes induced by BTP-001 on the metabolome, given the rapid response observed in the signalome. Nonetheless, the log_2_ fold-change (LFC) of the metabolite pools in the treated samples compared to the untreated control still show some changes (Figure 3C). However, PCA analysis of these pools revealed that mainly treatment with the high dose of JK-274 (7.5 μM) yielded a significant change in intracellular metabolite pools compared to the control, indicated by the respective 95% confidence regions not overlapping (Figure 3D).

#### 3.3.1. JK-274 and BTP-001 induce stress and virulence factors

**Figure 4:**
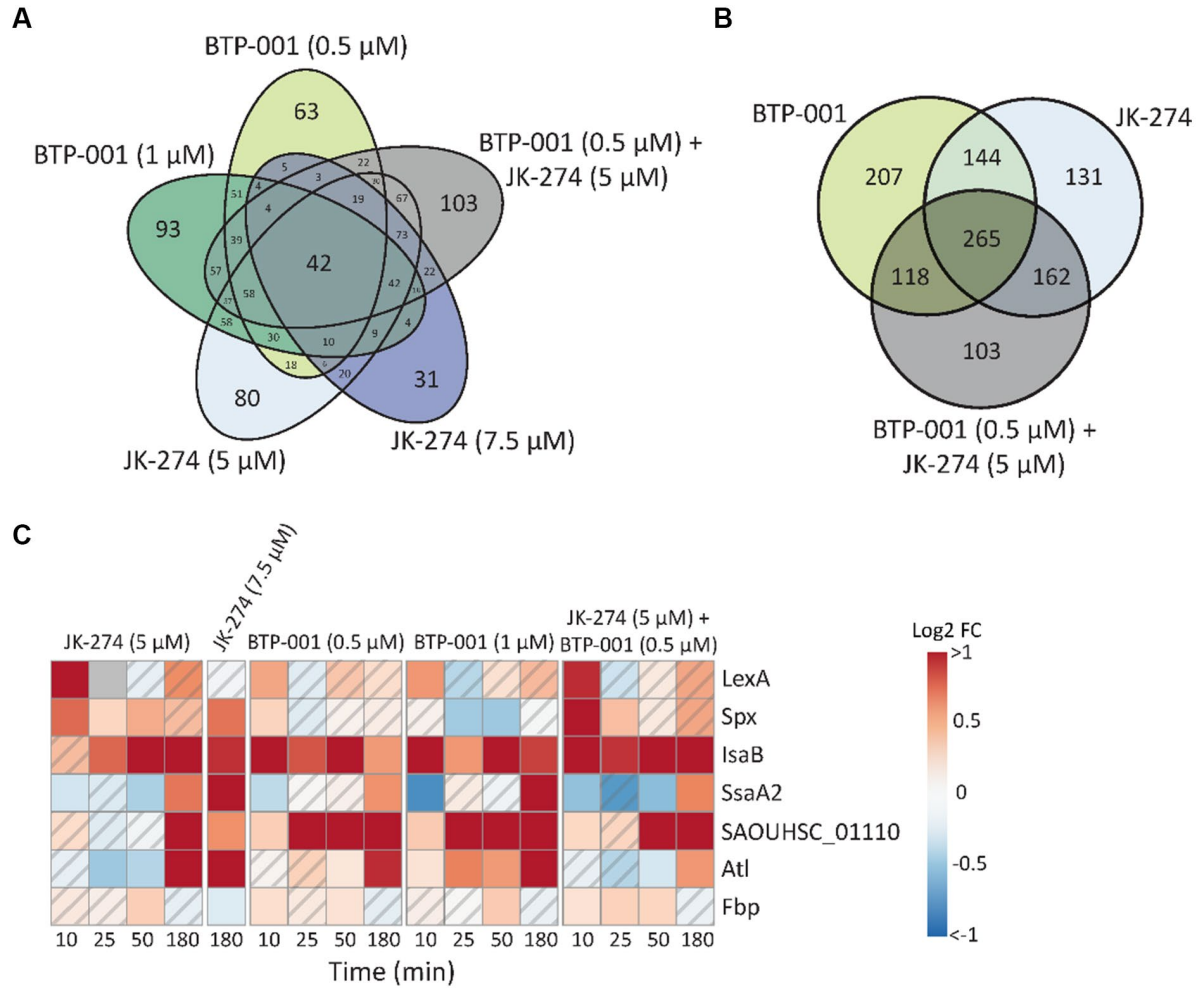
Many proteins have differential pulldown in all treatment groups. **(A)** Venn diagram of the number differentially pulled down DPD proteins that overlap between the 5 treatment groups. All timepoints have been merged. **(B)** Venn diagram of the number DPD proteins that overlap in which the low and high dose treatments of JK-274 and BTP-001, respectively, have been merged. All timepoints included. **(C)** A heatmap showing the log_2_ fold change (FC) in protein pulldown compared to the untreated control of selected proteins acting in bacterial virulence and cellular stress responses after treatment with JK-274 and BTP-001. A hatched field suggests a trend but lacks statistical significance.

Many stressors, including various antibiotics, may trigger similar responses in bacteria. Here, we found that 42 proteins exhibited DPD in all treatment groups (Figure 4A). When the high and low doses of JK-274 and BTP-001, respectively, were viewed as one treatment group, we identified over 265 DPD proteins shared by all treatment groups (Figure 4B). Some of the DPD proteins shared between treatment groups that are involved in stress responses are showed in Figure 4C. Activation of LexA, a transcriptional repressor of genes involved in the SOS response, increased in all treatment groups 10 min after treatment compared to the untreated control. Spx, another transcriptional regulator of global stress in *S. aureus* also showed increased pulldown in all treatments 10 min post-treatment. In addition, several other proteins which regulate or act as virulence factors in *S. aureus* showed DPD in all treatments, e.g., fibrinogen binding proteins like SAOUHSC_01110 and immune evasion proteins like IsaB, SsaA2, and Atl (Figure 4C).

All treatments increased pulldown of Fbp (fructose-1,6-bisphosphatase) at 50 min. Fbp is a key rate-limiting enzyme in gluconeogenesis that catalyzes the conversion of fructose-1,6-bisphosphate (F1,6B) to fructose-6-phosphate (F6P). Interestingly, all treatments also resulted in increased intracellular F6P pools 180 min post-treatments, although not significantly (Figure 3C). A known activator of Fbp is AMP, another metabolite with increased levels after treatment with both drugs (Figure 3). Treatment with bactericidal agents often leads to depletion of ATP in an effort to the repair damage, often yielding increased ADP and AMP pools (Belenky et al., 2015).

Overall, the differential pulldown of these proteins and change in metabolite pools collectively indicate that all treatment doses triggered a stress response without causing extensive bacterial cell death.

### 3.4. JK-274 reduces activity of TCA cycle enzymes and induces nitric oxide stress in *Staphylococcus aureus*

**Figure 5:**
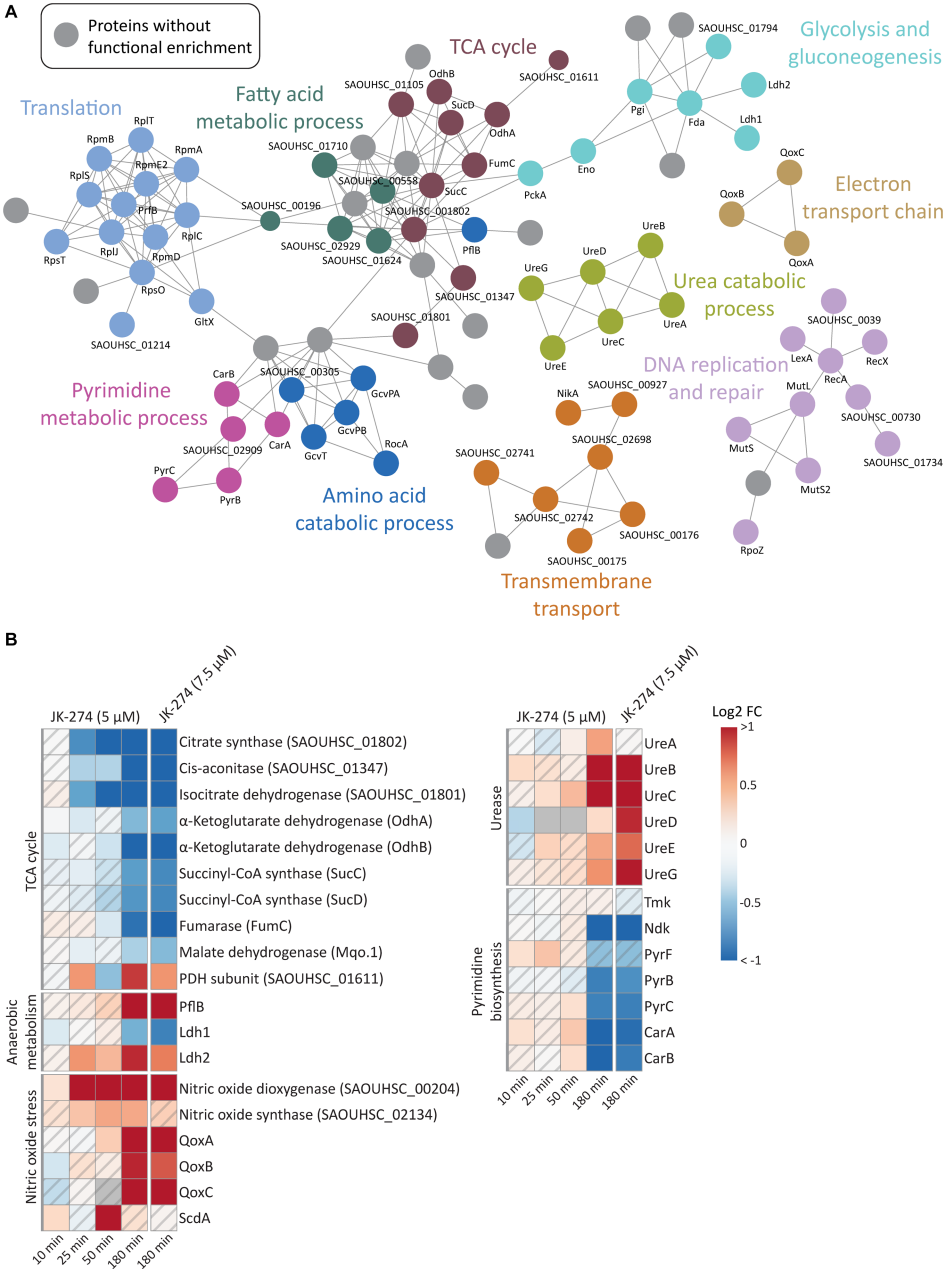
The effects of JK-274 on protein pulldown. **(A)** A STRING physical interaction network (confidence score > 0.9) of significant differentially pulled down (DPD) proteins in *S. aureus* according to the Wilcoxon signed rank test and moderated t-statistics (value of *p* ≤0.05). **(B)** Heatmap displaying the log_2_ fold change (FC) in pulldown for a select group of proteins in JK-274 treated *S. aureus* compared to an untreated control. Grey indicates no data. A hatched field suggests a trend but lacks statistical significance.

A gene ontology (GO) enrichment analysis of biological processes revealed that JK-274 led to DPD of proteins enriched for tricarboxylic acid (TCA) cycle enzymes, as visualized in a STRING physical interaction network (Figure 5A, burgundy circles). Of the 9 TCA cycle enzymes detected, 8 showed a significantly reduced pulldown at 50 and 180 min (Figure 5B). The first 3 enzymes in the TCA cycle also showed reduced pulldown at 25 min post-treatment. Additionally, pulldown of the proteins PflB (formate acetyltransferase) and Ldh2 (L-lactate dehydrogenase 2), which are associated with anaerobic and fermentative metabolism, increased after treatment (Figure 5B), possibly to compensate for reduced flow though the TCA cycle. Perturbations to the TCA cycle were supported by a decrease in intracellular levels of TCA cycle metabolites like α-ketoglutaric acid (α-KG) and malic acid (Mal), especially after treatment with the high dose of JK-274 (Figure 3C).

JK-274 treatment significantly increased pulldown of nitric oxide dioxygenase (NOD, SAOUHSC_00204), the protein that catalyzes the conversion of nitric oxide (NO) into nitrate and NADPH (Figure 5B). We detected an increase in NOD pulldown from 25 min post-treatment, corresponding to the decrease in TCA cycle enzymes. One of the most sensitive targets of NO is aconitase, which catalyzes the isomerization of citrate to isocitrate in the TCA cycle; thus, reduced pulldown of TCA cycle enzymes may be indirectly due to induction of NO stress or via direct inhibition. Microbes often reduce citrate flux into the TCA cycle under NO stress (Auger et al., 2011; Richardson et al., 2011; Gupta et al., 2012). Accordingly, the metabolic data showed a decrease in α-KG levels after JK-274 treatment (Figure 3C). Increased pulldown of Qox proteins and ScdA provided a second indication that JK-274 triggered NO stress as NO stress is shown to increase cytochrome biosynthesis (QoxA, QoxB, QoxC, and QoxD) and heme repair (ScdA) (Kinkel et al., 2013).

After treatment with JK-274, pulldown of urease subunits (UreA, UreB, UreC) and accessory proteins (UreD, UreE, UreG) increased (Figure 5B). Ureases catalyzes the hydrolysis of urea into ammonia and carbon dioxide, and urease activity has been associated with bacterial virulence (Mobley et al., 1995; Konieczna et al., 2012). Due to possible inactivation of TCA cycle enzymes by JK-274, the urea cycle may provide an alternate carbon source for the TCA cycle via glutamate (DeMars and Bose, 2018) to compensate for reduced activity of citrate synthase, cis-aconitase, and isocitrate dehydrogenase.

### 3.5. JK-274 reduces activity of nucleoside diphosphate kinase and pyrimidine biosynthetic proteins

Previously, JK-274 showed inhibitory activity toward *E. coli* Tmk in an enzymatic assay, and based on structural similarity to known Tmk inhibitors, we postulated that JK-274 also inhibits Tmk in *S. aureus* (Olsen et al., 2022). Here, Tmk was pulled down, but with no significant difference between JK-274 and the untreated control. Conversely, pulldown of the essential protein Ndk (nucleoside diphosphate kinase) was significantly reduced 180 min after treatment (Figure 5B), indicating a late-stage or secondary response as JK-274 affected growth before 180 min (Supplementary Figure S1). Furthermore, pulldown of several proteins in the pyrimidine biosynthesis pathway, including the highly regulated PyrB protein, was reduced (Figure 5B). This is also reflected in decreased intracellular CMP and UMP pools after JK-274 treatment (Figure 3C).

### 3.6. BTP-001 reduces FtsA pulldown

**Figure 6:**
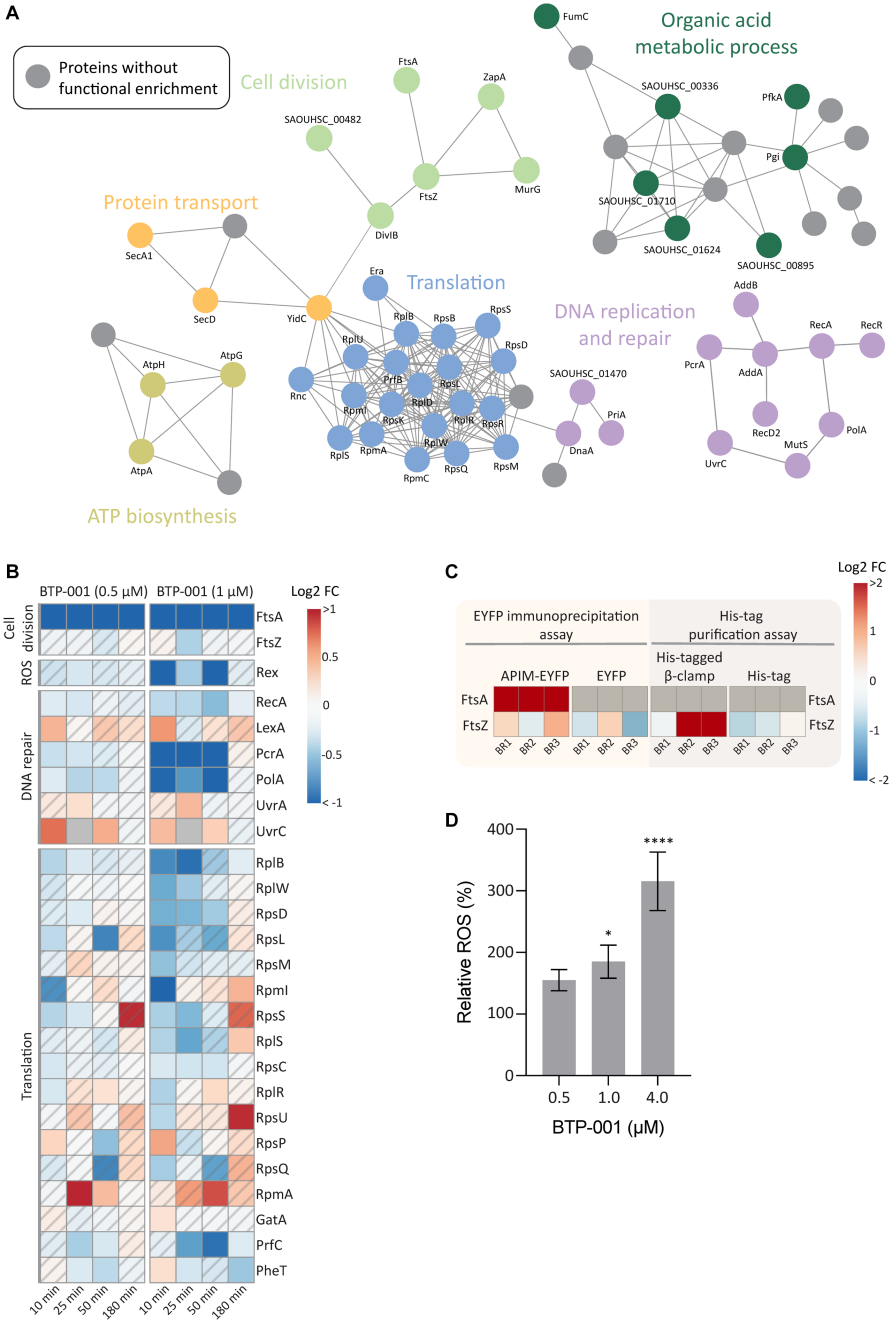
The effects of BTP-001 treatment. **(A)** A STRING physical interaction network (confidence score > 0.9) of significant differentially pulled down (DPD) proteins in *S. aureus* according to the Wilcoxon signed rank test and moderated t-statistics (value of *p* ≤0.05). **(B)** Heatmap displaying the log_2_ fold change (FC) for a select group of proteins in BTP-001 treated *S. aureus* compared to an untreated control. Grey indicates no data. A hatched field suggests a trend but lacks statistical significance. **(C)** A heatmap of the *E. coli* log_2_ transformed protein pulldown shown relative to the average across all samples for the EYFP immunoprecipitation assay (yellow) and His-tag purification assay (grey), respectively. FtsA was only detected for APIM-EYFP and set to max protein pulldown. Grey indicates no data. **(D)** Relative levels of intracellular ROS in *S. aureus* after treatment with BTP-001 compared to an untreated control. Mean ± SD, *n* = 3. One-way ANOVA (control vs. treatment): *p < 0.05, ****p < 0.0001.

A GO enrichment analysis of DPD proteins, as visualized in a STRING physical interaction network, showed cell division as a significantly enriched biological process after treatment with BTP-001 (Figure 6A, light green circles). The data revealed a strong reduction in pulldown of FtsA, an essential cell division protein that anchors the Z-ring, as the most significant effect of BTP-001 on the *S. aureus* signalome (Figure 6B; Supplementary Table S4). Even though *S. aureus* rapidly responded to BTP-001, a 10-fold reduction in FtsA pulldown was still detected 180 min after treatment. We also detected reduced pulled down of FtsZ, the protein that forms the Z-ring, 25 min after treatment with the high dose of BTP-001 (1 μM, Figure 6B). Interestingly, FtsA and FtsZ physically interacted with the APIM-motif and β-clamp, respectively, in parallel studies conducted in *E. coli*. Immunoprecipitation of the APIM-motif fused to EYFP (RWLVK-EYFP) from overexpressing *E. coli* pulled down FtsA from three independent BRs and from none of the EYFP-only controls (Figure 6C). This suggests that the APIM sequence (as in BTP-001) either directly or indirectly via the β-clamp binds to FtsA. FtsZ was also strongly pulled down from *E. coli* using a His-tagged β-clamp in 2 of 3 BRs, while much lower levels were pulled down in the corresponding control, indicating that the β-clamp and FtsZ likely bind or are in the same complex (Figure 6C). Together, these results indicate that the β-clamp might act as a scaffold in Z-ring formation and cell division, and that BTP-001 might disrupt this interaction.

FtsA is considered a potential antibiotic target as FtsA inhibition is shown to prevent Z-ring formation and promotes delocalization of FtsZ from the midcell (Mura et al., 2017; Ragunathan et al., 2019). In an attempt to correlate reduced FtsA pulldown with impaired Z-ring formation, we quantified Z-rings in BTP-001 (1 μM) treated and untreated *S. aureus*. However, no visual difference in Z-ring formation was detected between the treated and untreated cells at this dose (Supplementary Figure S2). Because BTP-001 did not completely prevent growth at this concentration, the data indicates Z-ring formation despite a substantial decrease in FtsA pulldown (Supplementary Figure S1). This suggests that FtsA levels were not critically low at this dose or that *S. aureus* has an unknown redundant protein or mechanism (Mura et al., 2017).

### 3.7. BTP-001 triggers oxidative stress in *Staphylococcus aureus*

High dose BTP-001 (1 μM) appeared to induce oxidative stress as we detected a strong reduction in the pulldown of Rex, a redox sensing transcriptional regulator (Figure 6B). Rex operates by binding NADH during oxidative stress and subsequently becoming deactivated and disassociating from the repressor site (Sickmier et al., 2005). Since the BTP-001 doses were below MIC, *S. aureus* was able to overcome the oxidative stress and at 180 min, Rex pulldown was the same as the control. Consistent with the potential role of ROS in the MoA, BTP-001 generated a dose-dependent increase in levels of intracellular ROS (Figure 6D). A common theory postulates that ROS formation plays a role in the MoA of most, if not all, bactericidal antibiotics (Dwyer et al., 2009, 2014). Other antimicrobial peptides have exhibited a higher anaerobic than aerobic MIC, indicating a central role of ROS formation in cell death (Choi et al., 2016, 2017). To test if ROS formation is critical for BTP-001 MoA, we performed an anaerobic MIC assay on *S. aureus*. However, MIC remained the same (data not shown). ROS might still contribute to BTP-001’s MoA as the 2-fold dilutions and 24-h incubation time used in the MIC assay cannot detect small changes and rapid effects.

### 3.8. BTP-001 affects activity of proteins involved in replication and translation

Based on previous work showing that APIM-peptides block replication and inhibit TLS at sub-MIC doses, we expected proteins associated with the replisome to be affected by treatment with BTP-001 (Nedal et al., 2020). Analysis of DPD proteins supported this as they showed increased pulldown of the DNA damage recognition proteins UvrA and UvrC, decreased pulldown of the DNA repair associated proteins PcrA (DNA helicase) and PolA (DNA polymerase), and lastly, reduced pulldown of RecA, the protein responsible for SOS induction by binding ssDNA (Figure 6B). Multiple proteins in DNA replication and DNA repair must interact with the DNA sliding clamp for optimal function (Moldovan et al., 2007). Reduced pulldown of PcrA and PolA could be due to BTP-001 blocking their direct interaction with the β-clamp, leading to protein destabilization and degradation. As expected, we mainly saw these changes with the high dose of BTP-001 which led to reduced growth compared to the control (Figure 2C; Supplementary Figure S1).

During growth, protein translation accounts for over 50% of the energy use in the bacterial cell (Russell and Cook, 1995). Therefore, many factors interact with the ribosome to tightly regulate protein synthesis in response to both internal and external stressors. Ten minutes after BTP-001 treatment, we detected 17 DPD proteins that belong to the cellular process translation (Figure 6A, blue circles), which consisted mostly of proteins from the large and small subunit of the 70S ribosome (Figure 6B). BTP-001 binds to the β-clamp at the same site as many replicative proteins and thereby stalls replications (Nedal et al., 2020). Therefore, a possible explanation for decreased ribosomal protein pulldown might be an increased degree of transcription-replication collision and thereby a collapse of translational complexes and degradation of these proteins (Nedal et al., 2020).

### 3.9. Increased stress responses likely contribute to the strong additive effect of BTP-001 and JK-274

**Figure 7:**
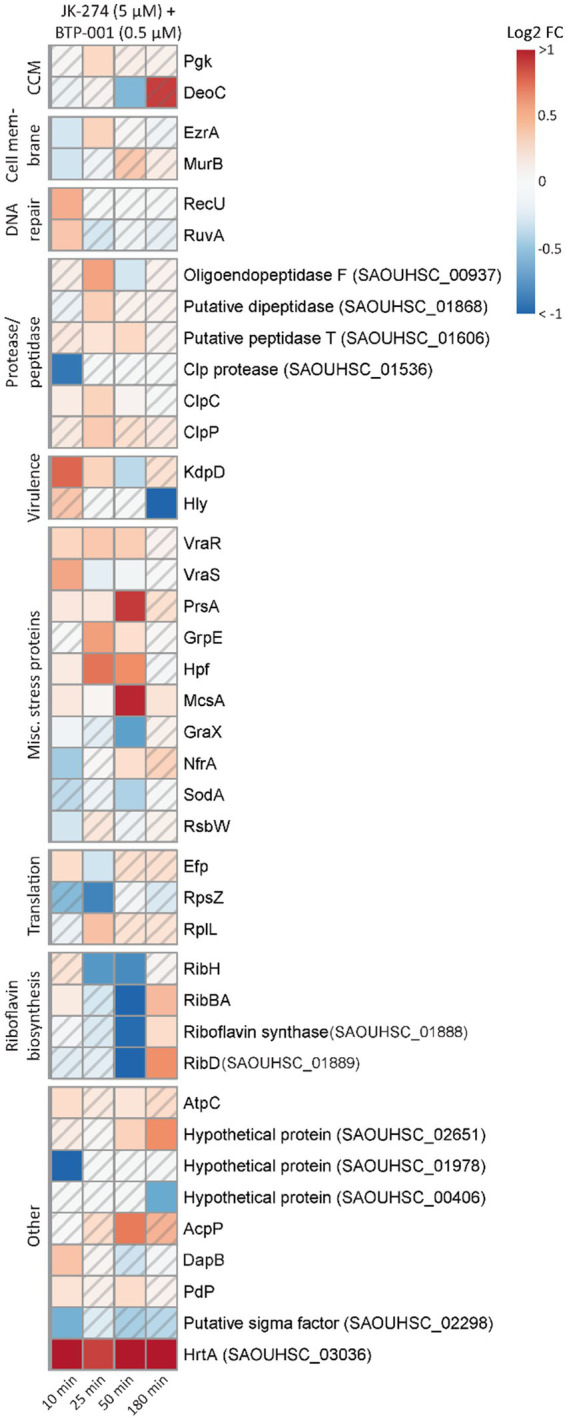
The unique effects of JK-274 and BTP-001 combination treatment. A heatmap displaying the log_2_ fold change (FC) in pulldown compared to the untreated control for a select group of proteins that were only significant for the combination treatment and not the single treatments. A hatched field suggests a trend but lacks statistical significance.

After combination treatment, the *S. aureus* signalome predominantly exhibited characteristics that were also observed after BTP-001 and JK-274 single treatment (Supplementary Figure S3). Although the signalome and metabolome data do not easily explain the strong additive effect, we observed that several DPD proteins involved in stress responses were only significant following combination treatment (Figures 4A, 7). These proteins include Holliday junction resolvases (RuvA and RecU), peptidases and proteases, virulence proteins (Hly and KdpD), and multiple other stress response proteins, e.g., the ribosomal hibernation protein (Hpf, inhibits translation), the PrsA foldase protein, and the metal-binding stress regulator McsA. This suggests that the combination treatment induces more stress than the high dose single treatments even though they had a similar growth rate. Additionally, at 50 min post-treatment, pulldown of riboflavin biosynthesis proteins was strongly reduced following combination treatment (Figure 7). Riboflavin is the precursor for flavin adenine dinucleotide (FAD) and flavin mononucleotide (FMN), both of which participate as cofactors in several redox reactions such as electron transfer, lipid metabolism, and oxidative stress response (Pinto and Zempleni, 2016). Reduced riboflavin biosynthesis might impact the ability of *S. aureus* to prevent and repair damage caused by NO (induced by JK-274) and ROS (induced by BTP-001) as many of the proteins involved in these processes require either FAD or FMN for activity (Chen et al., 2013; Ashoori and Saedisomeolia, 2014).

Overall, the data suggests that BTP-001 and JK-274 have very distinct MoAs that occur at different times, but when combined, lead to excessive stress. Perhaps, the bacterial adaptation toward one drug candidate is comprised of elements perturbed by the other. This could in many cases be an advantage to hamper resistance and to increase the efficacy.

### 3.10. Cytotoxicity of JK-274 and BTP-001 combination treatment in mammalian cells is low at doses required for bactericidal effect

**Figure 8:**
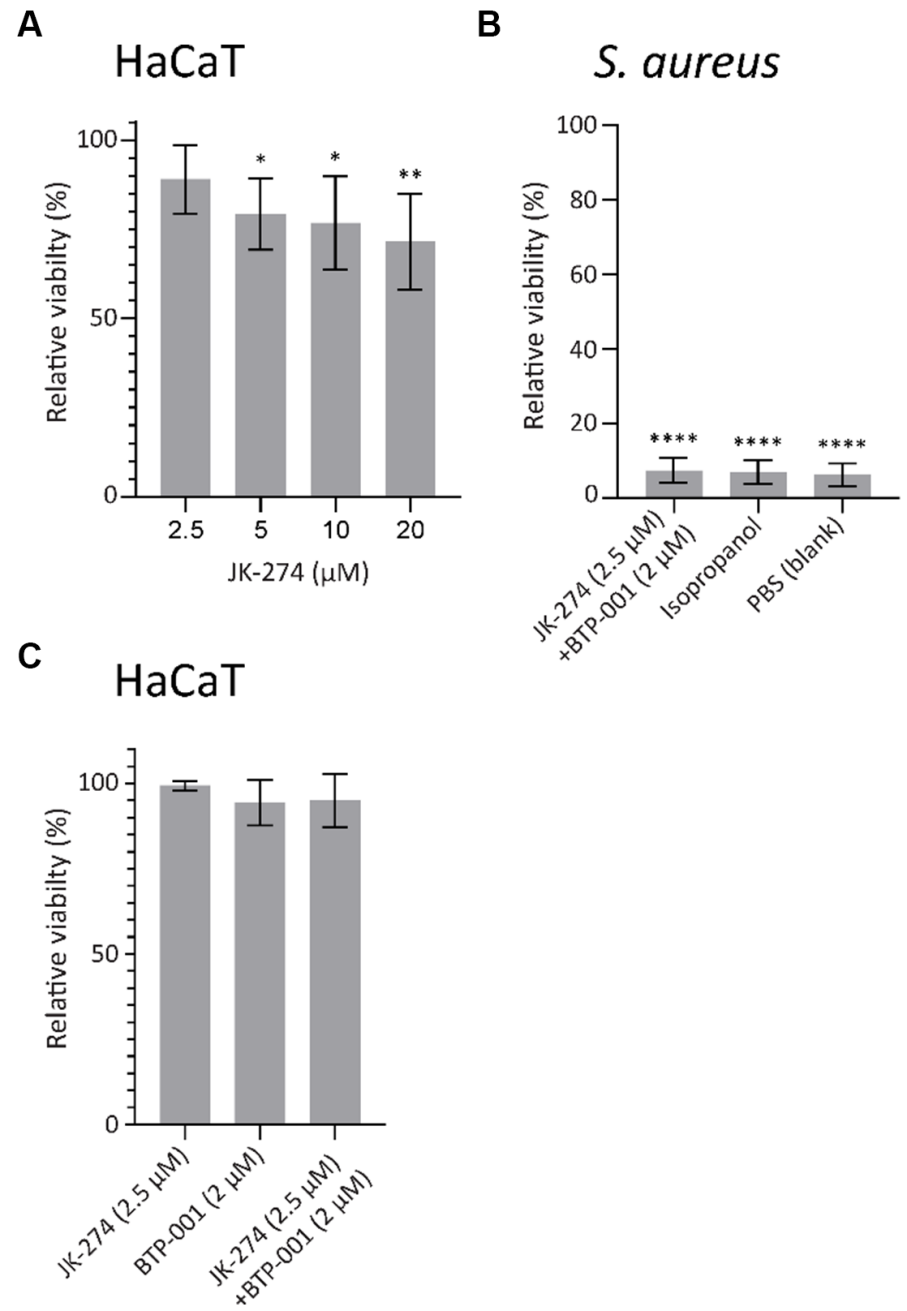
JK-274 and BTP-001 in combination show low cytotoxicity. **(A)** Relative viability of HaCaT cells following treatment with JK-274 (2.5–20 μM) compared to an untreated control measured after 24 h. Mean ± SD, *n* = 4. **(B)** Relative viability of *S. aureus* following treatment with JK-274 (2.5 μM) and BTP-001 (2 μM) combined and isopropanol relative to an untreated control measured after 4 h. Mean ± SD, *n* = 3. **(C)** Relative viability of HaCaT cells after treatment with JK-274 (2.5 μM) and BTP-001 (2 μM) relative to an untreated control. The media was exchanged for fresh media 4 h after treatment. Viability was measured the following day. Mean ± SD, *n* = 3. **(A,B)** *p < 0.05, **p < 0.01, ***p < 0.001, ****p < 0.0001, one-way ANOVA (control vs. treatment).

An antibiotic candidate should exhibit low or no toxicity toward mammalian cells at concentrations required to kill the host pathogen. To evaluate the toxicity of JK-274, we measured the viability of HaCaT cells, an immortalized human keratinocyte cell line, by measuring the fluorescence in a resazurin based viability assay. When treated for 24 h, 20 μM JK-274 decreased viability of the HaCaT cells by 35%; however, 2.5 μM JK-274, the dose needed for activity in combination with BTP-001, only reduced viability by 10% (Figure 8A). In *S. aureus*, combination treatment with JK-274 (2.5 μM) and BTP-001 (2 μM) reduced viability to the same level as the negative control (isopropanol, Figure 8B) after only 4 h and 100% lethality was confirmed by CFU assays (data not shown). Because the *in vivo* drug concentration is not constant over time, we also measured HaCaT cell viability after changing the media containing the treatment after 4 h. In this case, the combination dose, which was 100% bactericidal, did not affect cell viability after 24 h (Figure 8C). This shows that the cells tolerated the treatment; however, further modifications of JK-274 to increase specificity toward bacterial targets and reduce cytotoxicity is likely needed for use as a single agent.

## 4. Conclusion

To summarize, JK-274 reduces the activity of TCA cycle enzymes, possibly via nitric oxide induced stress. BTP-001 induces ROS, reduces levels of activated FtsA, and targets DNA replication and translation. Together, JK-274 and BTP-001 have a strong additive effect most likely due to the activation of several stress responses. JK-274 and BTP-001 showed no cytotoxicity toward HaCaT keratinocyte cells at treatment times and doses required for bactericidal effect. Thus, both drug candidates show potential and warrant further research.

## Data availability statement

The datasets presented in this study can be found in online repositories. The names of the repository/repositories and accession number(s) can be found in the article/Supplementary material.

## Author contributions

AS: Data curation, Formal analysis, Investigation, Methodology, Validation, Visualization, Writing – original draft, Writing – review and editing. OB: Methodology, Validation, Visualization, Writing – original draft, Writing – review and editing, Data curation, Formal analysis, Investigation. CS: Data curation, Formal analysis, Investigation, Methodology, Writing – review and editing. LR: Formal analysis, Investigation, Methodology, Validation, Writing – review and editing. CO: Investigation, Writing – review and editing. FHB: Investigation, Writing – review and editing. SR: Investigation, Methodology, and Writing – review and editing. FAB: Investigation, Writing – review and editing. ES: Writing – review and editing, Conceptualization, Supervision. BH: Conceptualization, Supervision, Writing – review and editing. PB: Conceptualization, Supervision, Writing – review and editing, Data curation. MO: Conceptualization, Supervision, Writing – review and editing, Data curation, Funding acquisition, Project administration.

## Funding

The author(s) declare financial support was received for the research, authorship, and/or publication of this article. This work was supported by NTNU Norwegian University of Science and Technology and Trond Mohn foundation. PROMEC is a member of the National Network of Advanced Proteomics Infrastructure (NAPI), which is funded by the RCN INFRASTRUKTUR-program (295910).The funders had no role in the study design, data collection and analysis, decision to publish, or preparation of the manuscript.

## Conflict of interest

The authors declare that the research was conducted in the absence of any commercial or financial relationships that could be construed as a potential conflict of interest.

## Publisher’s note

All claims expressed in this article are solely those of the authors and do not necessarily represent those of their affiliated organizations, or those of the publisher, the editors and the reviewers. Any product that may be evaluated in this article, or claim that may be made by its manufacturer, is not guaranteed or endorsed by the publisher.

## Figures and Tables

**Table 1:** 

ACN	Acetonitrile
AmBic	Ammonium bicarbonate
APIM	AlkB homolog 2 PCNA interacting motif
CAMHB	Cation-adjusted Mueller-Hinton broth
CCM	Central carbon metabolism
CFU	Colony forming unit
DPD	Differential pulldown
FAD	Flavin adenine dinucleotide
FAM	Flavin mononucleotide
GO	Gene ontology
IP	Immunoprecipitation
LFC	Log_2_ fold-change
LFQ	Label-free quantification
MIC	Minimal inhibitory concentration
MOA	Mode of action
NO	Nitric oxide
PCA	Principal component analysis
PCNA	Proliferating cell nuclear antigen
PPP	Pentose phosphate pathway
ROS	Reactive oxygen species
RT	Room temperature
TCA	Tricarboxylic acid
TLS	Translesion synthesis
