# Supplementary material for: Activation of multiple stress responses in Staphylococcus aureus substantially lowers the minimal inhibitory concentration when combining two novel antibiotic drug candidates
Source: Front Microbiol. 2023 Sep 25;14:1260120. doi: 10.3389/fmicb.2023.1260120 (PMC10564113; doi:10.3389/fmicb.2023.1260120)
Supplement: Supplementary file 1 [file Data_Sheet_1.zip › Supplementary figures and materials.PDF]

Supplementary Material

Supplementary Material

1 Figures

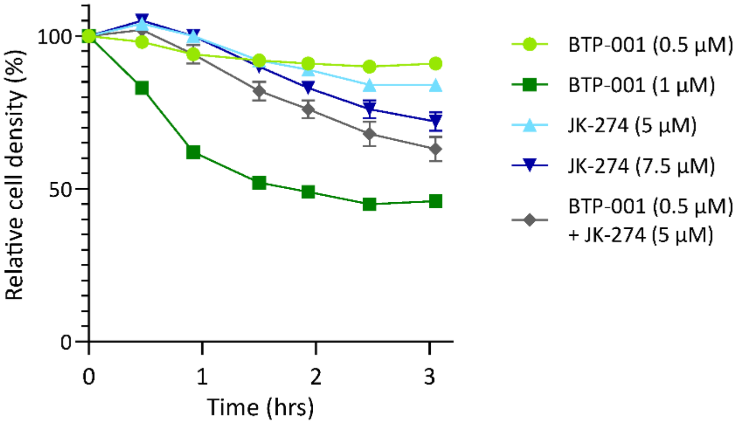

Supplementary Figure S1: Combination treatment yielded a lower growth rate than single treatment. Growth curve of *S. aureus* for 3 hours after treatment with JK-274 and BTP-001 single and combination treatment. Presented as relative cell density to the untreated control.

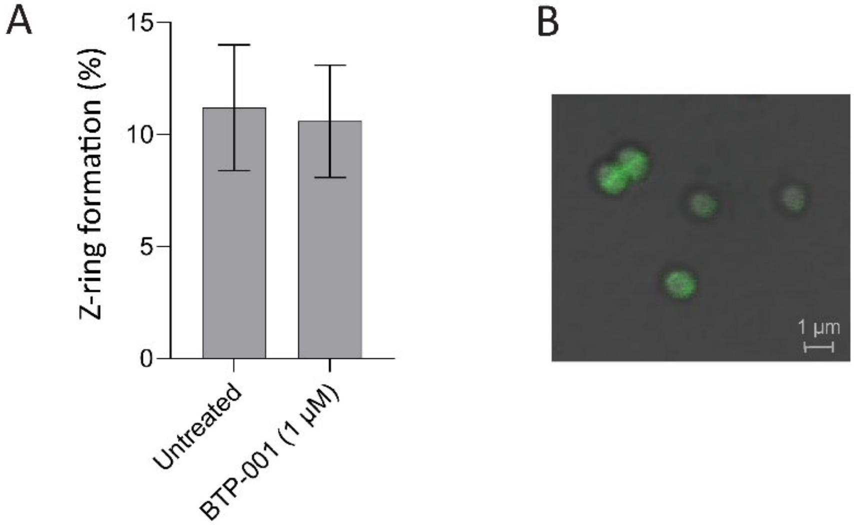

Supplementary Figure S2: BTP-001 (1µM) did not reduce Z-ring formation in *S. aureus*. (A) Proportion of *S. aureus* cells with a visually detectable Z-ring with and without BTP-001 treatment for 10 min. Mean ± SD; n=4. (B) Image of *S. aureus* treated with a FtsZ-binding fluorescent probe. Z-rings are present in the two cells in the upper left quadrant. Scale bar: 1 µm.

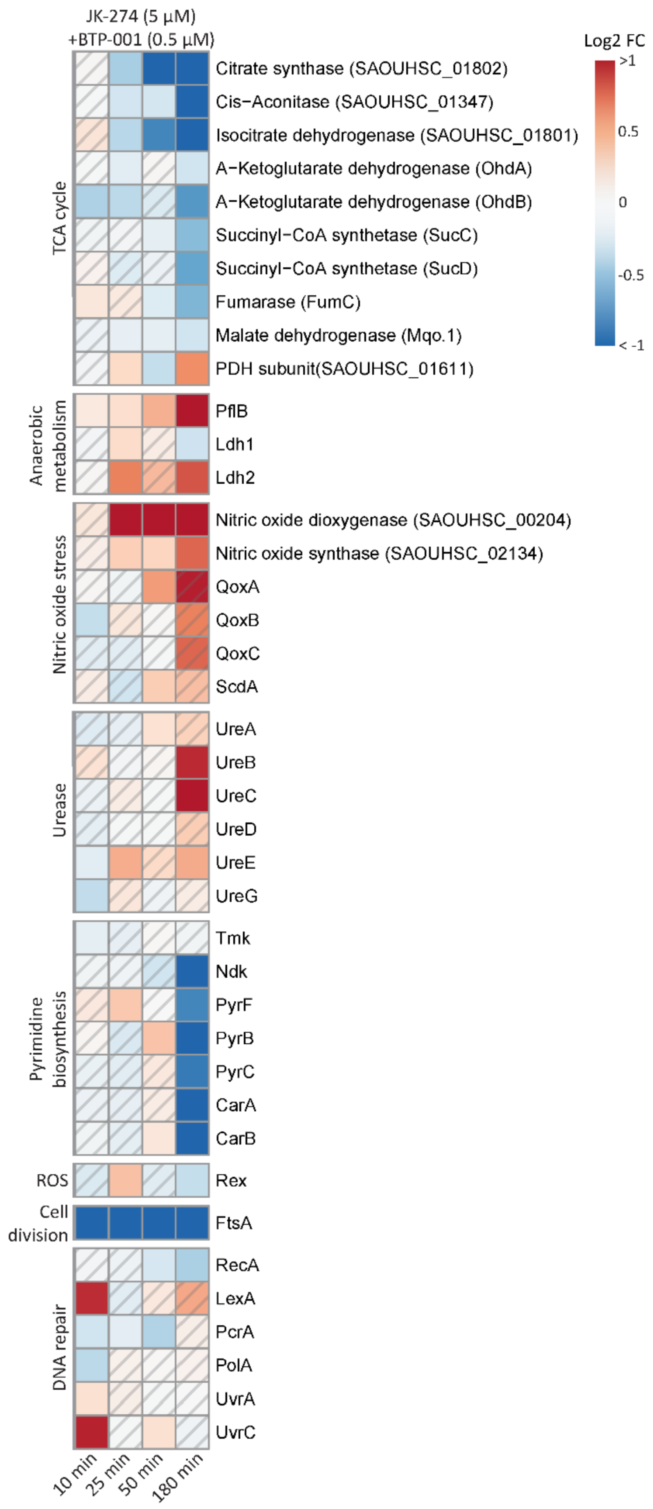

Supplementary Figure S3: The effects of JK-274 and BTP-001 combination treatment also seen in single treatment. A heatmap displaying the log2 fold change (FC) pulldown compared to the untreated control for a select group of proteins that were also significant for the single treatments. Hatched fields have a p-value > 0.1 and are not significant based on the Wilcoxon signed rank test.

2 Experimental scheme for L-1 and L-2

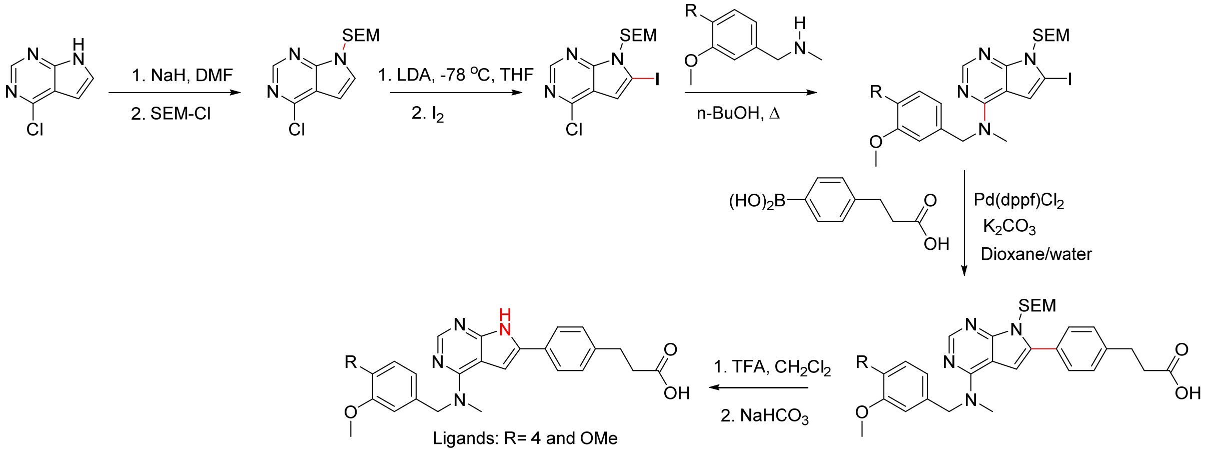

Supplementary Scheme S1: Synthetic route to the two new investigational ligands designed to mimic kinase inhibitors L-1 and L-2.

2.1 Experimental procedures

4-Chloro-7-((2-(trimethylsilyl)ethoxy)methyl)-7*H*-pyrrolo[2,3-*d*]pyrimidine

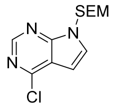

Dry DMF (50 mL) was cooled to 0 ˚C and added NaH (60% dispersion in oil) (2.70 g, 80.4 mmol). 4-Chloro-7*H*-pyrrolo[2,3-*d*]pyrimidine (10.2 g, 67.1 mmol) was dissolved in dry DMF (20 mL) and added portion-wise to the cooled suspension over 15 min. The reaction mixture was left stirring at 0 ˚C for 20 min. Then, SEM-Cl (14.2 mL, 80.4 mmol) was added. The mixture was left stirring for 1 h, while cooling, before quenching with sat. aq. NH_4Cl (2 mL). The mixture was transferred to a round-bottom flask and concentrated in vacuum. The concentrated residue was partitioned between CH_2Cl_2 (40 mL) and water (50 mL). The layers were separated, and the water-phase extracted with more CH_2Cl_2 (3 × 50 mL). The combined organic layers were washed with water (4 × 50 mL) and brine (50 mL), dried with anhydrous Na_2SO_4, filtered and concentrated in vacuo. The residue was purified by column chromatography on silica-gel (*n*-pentane/EtOAc 10:1, R_f = 0.24) yielding 14.3 g (50.3 mmol, 76%) of a clear oil. ^{1}H NMR (400 MHz, DMSO-*d6*) δ 8.68 (s, 1H), 7.87 (d, J = 3.6 Hz, 1H), 6.71 (d, J = 3.6 Hz, 1H), 5.65 (s, 2H), 3.52 (t, J = 8.0 Hz, 2H), 0.82 (t, J = 8.0 Hz, 2H), -0.10 (s, 9H); ^{13}C NMR (100 MHz, DMSO-*d6*) δ 151.3, 150.8, 150.7, 131.5, 116.9, 99.3, 72.9, 65.8, 17.1, -1.5 (3C); IR (neat, cm^{-1}): 3120 (br,w), 3088 (w), 2950 (m), 2896 (w), 1587 (s), 1542 (s), 1348 (s), 1033 (s), 833 (s), 744 (s); HRMS (ES+, m/z): found 284.099, calcd. $C_{12}H_{19}N_3OSiCl$ $[M+H]^+$, 284.0986.

4-Chloro-6-iodo-7-((2-(trimethylsilyl)-ethoxy)methyl)-7*H*-pyrrolo[2,3-*d*]pyrimidine (2)

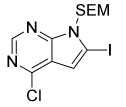

Under an N_2 atmosphere 4-chloro-7-((2-(trimethylsilyl)-ethoxy)methyl)-7*H*-pyrrolo[2,3-*d*]pyrimidine (5.00 g, 17.6 mmol) was dissolved in dry THF (70 mL) and cooled down to -78 °C. Then, LDA (2 M in THF/*n*-hexane/ethylbenzene, 13.3 mL, 26.6 mmol) was added drop-wise over 30 min. This was followed by drop wise addition of I_2 (5.08 g, 20.1 mmol) dissolved in THF (12 mL). After another 30 min, the reaction mixture was quenched with saturated NH_4Cl solution (0.5 mL) and stirred until ambient was reached. The mixture was concentrated and diluted with 10% $Na_2S_2O_3$ solution (20 mL), CH_2Cl_2 (25 mL) and water (30 mL). After phase separation, the water phase was extracted with more CH_2Cl_2 (4 × 20 mL). The combined organic phase was dried over Na_2SO_4 and the solvent was removed under reduced pressure. The crude product was purified by silica-gel flash chromatography (*n*-pentane/EtOAc - 9:1, R_f = 0.44) giving 6.60 g (16.1 mmol, 92%) of 4-chloro-6-iodo-7-((2-(trimethylsilyl)-ethoxy)methyl)-7*H*-pyrrolo[2,3-*d*]pyrimidine as a grey powder, mp. 99 - 101 °C; ^{1}H NMR (600 MHz, DMSO-d_6) δ 8.62 (s, 1H), 7.11 (s, 1H), 5.61 (s, 2H), 3.53, (t, J = 7.9 Hz, 2H), 0.82 (t, J = 7.9 Hz, 2H), -1.11 (s, 9H); ^{13}C NMR (150 MHz, DMSO-d_6) δ 152.5, 150.8, 149.0, 118.6, 109.8, 91.5, 73.5, 66.0, 17.1, -1.4 (3C); HRMS (ASAP-TOF, m/z): found 409.9951, calcd. $C_{12}H_{19}N_3OSiCl$ $[M+H]^+$, 409.9952.

6-Iodo-N-(4-methoxybenzyl)-N-methyl-7-((2-(trimethylsilyl)ethoxy)methyl)-7H-pyrrolo[2,3-d]pyrimidin-4-amine

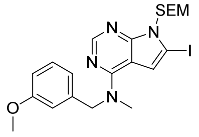

4-Chloro-6-iodo-7-((2-(trimethylsilyl)-ethoxy)methyl)-7*H*-pyrrolo[2,3-*d*]pyrimidine (234 mg, 0.57 mmol) was dissolved in dry *n*-BuOH (6 mL) followed by addition of 1-(3-methoxyphenyl)-*N*-methylmethanamine (265 mg, 1.71 mmol). The reaction was stirred at 140 ˚C for 17 h. Following evaporation of solvent, the mixture was extracted with water (20 mL) and CH_2Cl_2 (20 mL). After phase separation, the aqueous phase was extracted with more CH_2Cl_2 (3 × 20 mL) and the combined organic layers were washed with brine (20 mL) before being dried over Na_2SO_4, filtered and concentrated in vacuo. Purification by silica-gel chromatography (*n*-pentane/EtOAc, 4:1, R_f = 0.34) gave 276 mg (0.53 mmol, 93%) of a brown oil. ^{1}H NMR (600 MHz, DMSO-d_6) δ 8.14 (s, 1H), 7.23 (t, J = 7.8 Hz, 1H), 6.94 (s, 1H), 6.82 – 6.78 (m, 3H), 5.50 (s, 2H), 4.96 (s, 2H), 3.70 (s, 3H), 3.55 – 3.49 (m, 2H), 3.29 (s, 3H), 0.85 – 0.78 (m, 2H), -0.09 (s, 9H); ^{13}C NMR (151 MHz, DMSO-d_6) δ 159.4, 155.4, 152.8, 151.2,

139.7, 129.7, 118.9, 112.8, 112.2, 112.1, 104.1, 80.4, 72.7, 65.5, 54.9,52.7, 37.4, 17.1, -1.4 (3C); HRMS (ES+, m/z): found 525.1182, calcd. $C_{21}H_{30}IN_4O_2Si$ $[M+H]^+$, 525.1183.

N-(3,4-Dimethoxybenzyl)-6-iodo-N-methyl-7-((2-(trimethylsilyl)ethoxy)methyl)-7H-pyrrolo[2,3-d]pyrimidin-4-amine

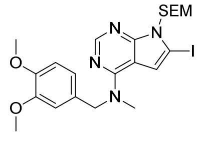

4-Chloro-6-iodo-7-((2-(trimethylsilyl)-ethoxy)methyl)-7*H*-pyrrolo[2,3-*d*]pyrimidine (299 mg, 0.73 mmol) was dissolved in dry *n*-BuOH (6 mL) followed by addition of 3,4-dimethoxyphenyl-N-methylmethanamine (396 mg, 2.19 mmol). The reaction was stirred at 140 ˚C for 17 h. Following evaporation of solvent, the mixture was extracted with water (20 mL) and CH_2Cl_2 (20 mL). After phase separation, the aqueous phase was extracted with more CH_2Cl_2 (3 × 20 mL) and the combined organic layers were washed with brine (20 mL) before being dried over Na_2SO_4, filtered and concentrated in vacuo. Purification by silica-gel chromatography (*n*-pentane/EtOAc, 3:1, R_f = 0.54) gave 382 mg (0.69 mmol, 95%) of a brown oil. ^{1}H NMR (600 MHz, DMSO-*d*$_6$) δ 8.15 (s, 1H), 6.94 (s, 1H), 6.91 – 6.89 (m, 2H), 6.72 (dd, *J* = 8.2, 2.0 Hz, 1H), 5.49 (s, 2H), 4.90 (s, 2H), 3.71 (s, 3H), 3.68 (s, 3H), 3.52 (dd, *J* = 8.4, 7.5 Hz, 2H), 3.27 (s, 3H), 0.85 – 0.79 (m, 2H), -0.09 (s, 9H); ^{13}C NMR (151 MHz, DMSO-*d*$_6$) δ 155.4, 152.7, 151.2, 148.8, 147.9, 130.3, 118.9, 112.3, 111.9, 111.1, 104.1, 80.3, 72.7, 65.5, 55.5, 55.4, 52.4, 37.1, 17.1, -1.4 (3C); HRMS (ES+, m/z): found 555.1296 calcd. $C_{22}H_{32}IN_4O_3Si$ $[M+H]^+$, 555.1288.

3-(4-(4-((4-Methoxybenzyl)(methyl)amino)-7-((2-(trimethylsilyl)ethoxy)methyl)-7H-pyrrolo[2,3-d]pyrimidin-6-yl)phenyl)propanoic acid

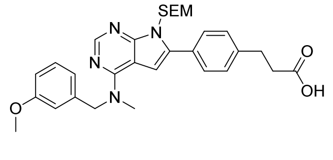

6-Iodo-*N*-(4-methoxybenzyl)-N-methyl-7-((2-(trimethylsilyl)ethoxy)methyl)-7*H*-pyrrolo[2,3-*d*]pyrimidin-4-amine (194 mg, 0.36 mmol), 4-(2-carboxyethyl)benzeneboronic acid (83 mg, 0.43 mmol), K_2CO_3 (151 mg, 1.08 mmol) and Pd(dppf)Cl_2 (13.1 mg, 19.0 µmol) were added to a Schlenk tube in an N_2 atmosphere. Degassed H_2O (2 mL) and 1,4-dioxane (4 mL) were added, and the reaction was stirred at 80 °C for 5 min. The vessel was then cooled to room temperature before the addition of H_2O (20 mL) and CH_2Cl_2 (20 mL). After phase separation, the aqueous phase was extracted with more CH_2Cl_2 (3 × 20 mL) and the combined organic layers were washed with brine (20 mL) before being dried over Na_2SO_4, filtered and concentrated in vacuo. Purification by silica-gel chromatography (*n*-pentane/EtOAc/AcOH, 125:75:1, R_f = 0.31) gave 139 mg (0.26 mmol, 72%) of a clear oil. ^{1}H NMR (600 MHz, DMSO-*d*$_6$) δ 8.23 (s, 1H), 7.61 (d, *J* = 8.1 Hz, 2H), 7.32 (d, *J* = 8.2 Hz, 2H), 7.23 (dd, *J* =

9.2, 7.2 Hz, 1H), 6.81 (d, *J* = 6.6 Hz, 3H), 6.75 (s, 1H), 5.52 (s, 2H), 5.01 (s, 2H), 3.70 (s, 3H), 3.63 – 3.55 (m, 2H), 3.35 (s, 3H), 2.86 (t, *J* = 7.6 Hz, 2H), 2.57 (t, *J* = 7.6 Hz, 2H), 0.87 – 0.79 (m, 2H), -0.10 (s, 9H); ^{13}C NMR (151 MHz, DMSO-*d6*) δ 173.9, 159.5, 156.5, 153.1, 151.3, 141.1, 140.0, 136.5, 129.8, 129.3, 128.7 (2C), 128.6 (2C), 119.1, 112.9, 112.2, 102.2, 102.1, 70.4, 65.8, 55.0, 52.9, 37.6, 35.1, 30.1, 17.4, -1.3 (3C); HRMS (ES+, m/z): found 547.2742, calcd. $C_{30}H_{39}N_4O_4Si$ $[M+H]^+$, 547.2741.

3-(4-(4-((3,4-dimethoxybenzyl)(methyl)amino)-7-((2-(trimethylsilyl)ethoxy)methyl)-7H-pyrrolo[2,3-d]pyrimidin-6-yl)phenyl)propanoic acid

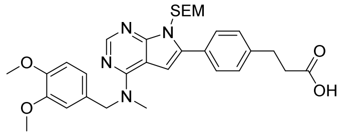

N-(3,4-Dimethoxybenzyl)-6-iodo-*N*-methyl-7-((2-(trimethylsilyl)ethoxy)methyl)-7*H*-pyrrolo[2,3-d]pyrimidin-4-amine (250 mg, 0.45 mmol), 4-(2-carboxyethyl)benzeneboronic acid (105 mg, 0.54 mmol), K_2CO_3 (187 mg, 1.35 mmol) and Pd(dppf)Cl_2 (16.5 mg, 22.5 µmol) were added to a Schlenk tube in an N_2 atmosphere. Degassed H_2O (2 mL) and 1,4-dioxane (4 mL) were added, and the reaction was stirred at 80 °C for 5 min. The vessel was then cooled to room temperature before the addition of H_2O (20 mL) and CH_2Cl_2 (20 mL). After phase separation, the aqueous phase was extracted with more CH_2Cl_2 (3 × 20 mL) and the combined organic layers were washed with brine (20 mL) before being dried over Na_2SO_4, filtered and concentrated in vacuo. Purification by silica-gel chromatography (*n*-pentane/EtOAc/AcOH, 1:1:0.01, R_f = 0.25) gave 168 mg (0.29 mmol, 64%) of a white waxy solid. ^{1}H NMR (600 MHz, DMSO-*d6*) δ 12.14 (s, 1H), 8.25 (s, 1H), 7.62 (d, *J* = 8.2 Hz, 2H), 7.41 – 7.24 (m, 2H), 6.94 (d, *J* = 2.0 Hz, 1H), 6.88 (d, *J* = 8.3 Hz, 1H), 6.76 (dd, *J* = 8.2, 2.0 Hz, 2H), 5.53 (s, 2H), 4.96 (s, 2H), 3.70 (s, 3H), 3.69 (s, 3H), 3.63 – 3.58 (m, 2H), 3.33 (s, 3H), 2.86 (t, *J* = 7.6 Hz, 2H), 2.61 – 2.55 (m, 2H), 0.84 – 0.80 (m, 2H), -0.09 (s, 9H); ^{13}C NMR (151 MHz, DMSO-*d6*) δ 173.7, 156.4, 153.0, 151.2, 148.8, 147.9, 140.9, 136.3, 130.5, 129.2, 128.6 (2C), 128.5 (2C), 119.1, 111.9, 111.2, 102.2, 102.1, 70.3, 65.7, 55.5, 55.4, 52.5, 37.2, 35.0, 30.0, 17.3, -1.4 (3C); HRMS (ES+, m/z): found 577.2842, calcd. $C_{31}H_{41}N_4O_5Si$ $[M+H]^+$, 577.2846.

3-(4-(4-((3-methoxybenzyl)(methyl)amino)-7H-pyrrolo[2,3-d]pyrimidin-6-yl)phenyl)propanoic acid (Ligand 1, L-1)

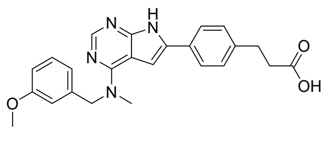

3-(4-(4-((4-Methoxybenzyl)(methyl)amino)-7-((2-(trimethylsilyl)ethoxy)methyl)-7*H*-pyrrolo[2,3-*d*]pyrimidin-6-yl)phenyl)propanoic acid (52 mg, 0.09 mmol) was stirred in TFA (1 mL) and CH_2Cl_2 (5 mL) at 50 °C for 3 h. The reaction mixture was then concentrated in *vacuo* before addition of THF (5 mL) and $NaHCO_3$(sat) (5 mL). The mixture was then stirred for 16 h at 21°C.The reaction mixture was concentrated in *vacuo*, and added CH_2Cl_2 (25 mL) and MeOH (5 mL) before being stirred for 2 h. The mixture was filtrated through celite and concentrated in vacuo. Purification by silica-gel chromatography (CH_2Cl_2/MeOH/AcOH, 97:3:1, R_f = 0.32) gave 23 mg (0.06 mmol, 67%) of an off white powder, mp. 208 – 213 °C; ^{1}H NMR (400 MHz, DMSO-d_6) δ 12.13 (d, *J* = 2.1 Hz, 1H), 8.14 (s, 1H), 7.75 (d, *J* = 8.3 Hz, 2H), 7.30 – 7.20 (m, 3H), 6.99 (d, *J* = 2.0 Hz, 1H), 6.86 – 6.78 (m, 3H), 5.01 (s, 2H), 3.71 (s, 3H), 3.36 (s, 3H), 2.83 (t, *J* = 7.5 Hz, 2H), 2.54 (t, *J* = 7.6 Hz, 2H). ^{13}C NMR (101 MHz, DMSO-d_6) δ 172.1, 159.4, 156.4, 152.9, 151.0, 140.2, 133.3, 129.6, 129.5, 129.3, 128.7 (2C), 124.7 (2C), 119.1, 112.9, 112.0, 103.3, 98.4, 54.94, 52.6, 37.4, 35.1, 30.1. IR (neat, cm^{-1}): 3189 (br,w), 2958 (br,w), 2839 (w), 1709 (s), 1548 (m), 1515 (m), 1415 (m), 1252(s), 1146 (s), 920 (s), 788 (s); HRMS (ES+, m/z): found 417.1927, calcd. $C_{24}H_{25}N_4O_3$ $[M+H]^+$, 417.1927.

3-(4-(4-((3,4-dimethoxybenzyl)(methyl)amino)-7H-pyrrolo[2,3-d]pyrimidin-6-yl)phenyl)propanoic acid (Ligand 2, L-2)

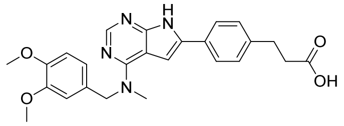

3-(4-(4-((3,4-Dimethoxybenzyl)(methyl)amino)-7-((2-(trimethylsilyl)ethoxy)methyl)-7*H*-pyrrolo[2,3-*d*]pyrimidin-6-yl)phenyl)propanoic acid (54 mg, 0.09 mmol) was stirred in TFA (1 mL) and CH_2Cl_2 (5 mL) at 50 °C for 2 h. The reaction mixture was then concentrated in *vacuo* before addition of THF (5 mL) and $NaHCO_3$ (5 mL, sat.). The mixture was then stirred for 16 h at 21°C.The reaction mixture was concentrated in *vacuo*, and added CH_2Cl_2 (25 mL) and MeOH (5 mL) before being stirred for 2 h. The mixture was filtrated through celite and concentrated in vacuo. Purification by silica-gel chromatography (CH_2Cl_2/MeOH/AcOH, 95:5:1, R_f = 0.51) resulted in 22 mg (0.05 mmol, 56%) of an off-white powder, mp. 215 – 219 °C; ^{1}H NMR (600 MHz, DMSO-d_6) δ 12.13 (s, 1H), 8.16 (s, 1H), 7.76 (d, *J* = 8.2 Hz, 2H), 7.27 (d, *J* = 8.2 Hz, 2H), 7.00 (s, 1H), 6.96 (d, *J* = 2.0 Hz, 1H), 6.89 (d, *J* = 8.3 Hz, 1H), 6.78 (dd, *J* = 8.2, 2.0 Hz, 1H), 4.96 (s, 2H), 3.71 (s, 3H), 3.71 (s, 3H), 3.34 (s, 3H), 2.83 (t, *J* = 7.6 Hz, 2H), 2.55 (t, *J* = 7.6 Hz, 2H); ^{13}C NMR (151 MHz, DMSO-d_6) δ 173.9, 156.4, 152.9, 151.0, 148.8, 147.9, 140.2, 133.2, 130.7, 129.3, 128.7 (2C), 124.7 (2C), 119.1, 111.9, 111.3, 103.3, 98.4, 55.4 (2C), 52.3, 37.2, 35.2, 30.1; IR (neat, cm^{-1}): 2997 (br,w), 2927 (br,w), 2834 (w), 1716 (s), 1624 (m), 1514 (m), 1411 (m), 1364 (m), 1263(s), 1194 (s), 919 (s), 784 (s), 640 (s); HRMS (ES+, m/z): found 447.2039, calcd. $C_{25}H_{27}N_4O_4$ $[M+H]^+$, 447.2032

2.2 NMR spectra

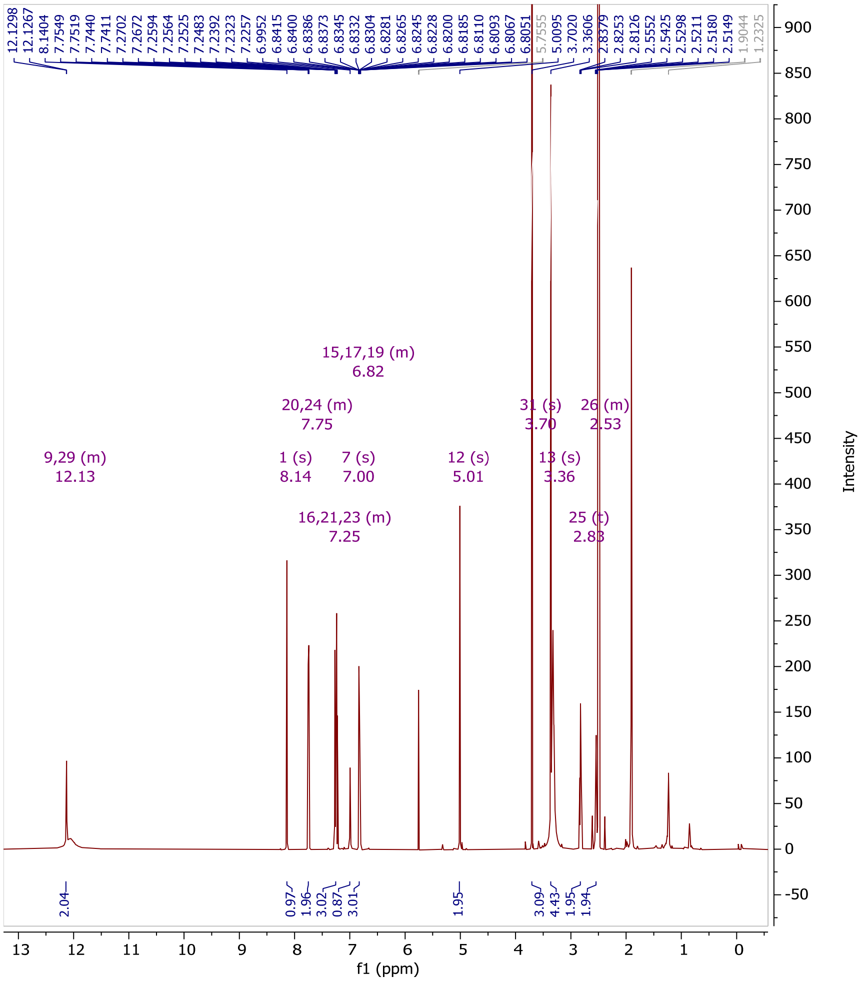

	Parameter	Value
1	Data File Name	F:/ NMR råfiler/ fab-02-78-2-/ 1/ fid
2	Title	fab-02-78-2-test.1.fid
3	Comment	PROTON DMSO {C:\ Users\ nmrsu\ Documents} Frithjob 7
4	Origin	Bruker BioSpin GmbH
5	Owner	nmrsu
6	Site	
7	Instrument	spect
8	Author	
9	Solvent	DMSO
10	Temperature	298.0
11	Pulse Sequence	zg30
12	Experiment	1D
13	Probe	Z117768_0061 (CP TCI 600S3 H-C/ N-D-05 Z)
14	Number of Scans	32
15	Receiver Gain	12.9
16	Relaxation Delay	1.0000
17	Pulse Width	8.0000
18	Presaturation Frequency	
19	Acquisition Time	2.7263
20	Acquisition Date	2023-05-09T16:54:51
21	Modification Date	2023-05-09T16:54:52
22	Class	
23	Spectrometer Frequency	600.23
24	Spectral Width	12019.2
25	Lowest Frequency	-2303.2
26	Nucleus	1H
27	Acquired Size	32768
28	Spectral Size	131072
29	Digital Resolution	0.09

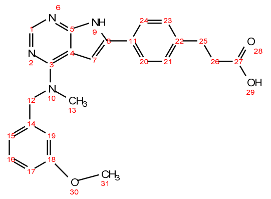

^{1}H NMR (600 MHz, DMSO-d_6) of L-1

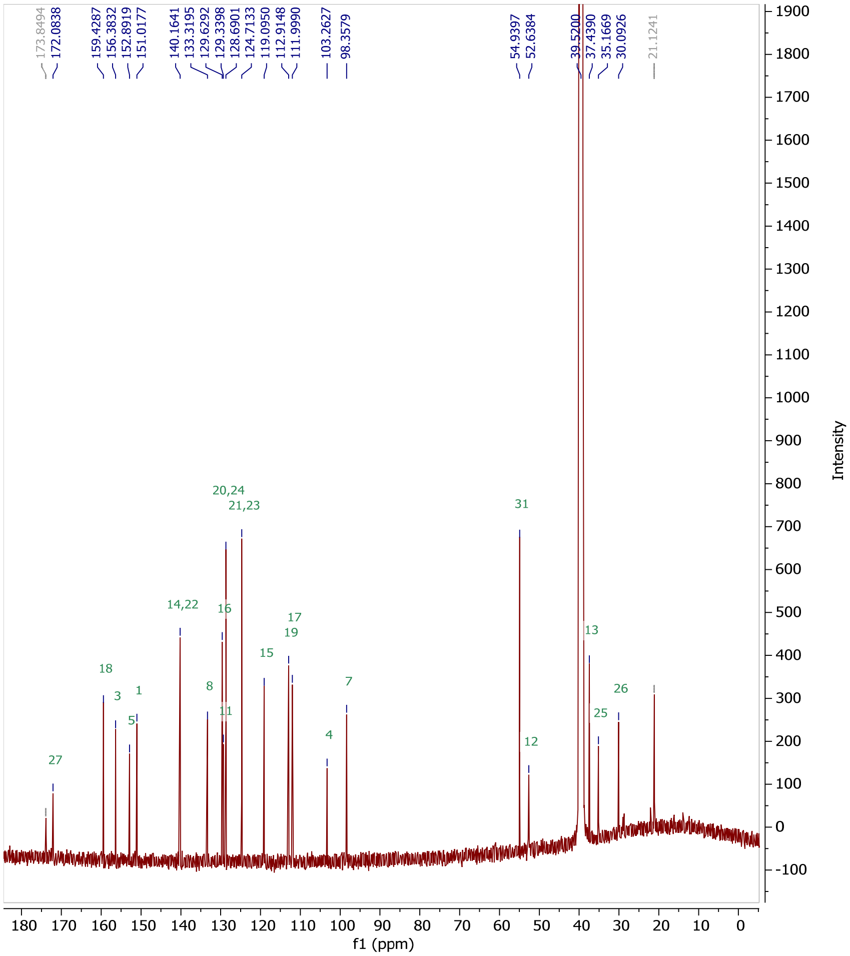

	Parameter	Value
1	Data File Name	F:/ / NMR råfiler/ fab-02-78-2/ 2/ fid
2	Title	fab-02-78-2-test.2.fid
3	Comment	C13CPD DMSO {C:\ Users\ nmrsu\ Documents} Frithjob 7
4	Origin	Bruker BioSpin GmbH
5	Owner	nmrsu
6	Site	
7	Instrument	spect
8	Author	
9	Solvent	DMSO
10	Temperature	298.0
11	Pulse Sequence	zgpg30
12	Experiment	1D
13	Probe	Z117768_0061 (CP TCI 600S3 H-C/ N-D-05 Z)
14	Number of Scans	5000
15	Receiver Gain	197.1
16	Relaxation Delay	2.0000
17	Pulse Width	11.4000
18	Presaturation Frequency	
19	Acquisition Time	0.9044
20	Acquisition Date	2023-05-09T21:02:36
21	Modification Date	2023-05-09T21:02:36
22	Class	
23	Spectrometer Frequency	150.94
24	Spectral Width	36231.9
25	Lowest Frequency	-3023.1
26	Nucleus	13C
27	Acquired Size	32768
28	Spectral Size	65536
29	Digital Resolution	0.55

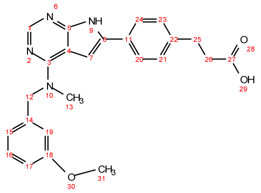

^{13}C NMR (150 MHz, DMSO-d_6) of L-1

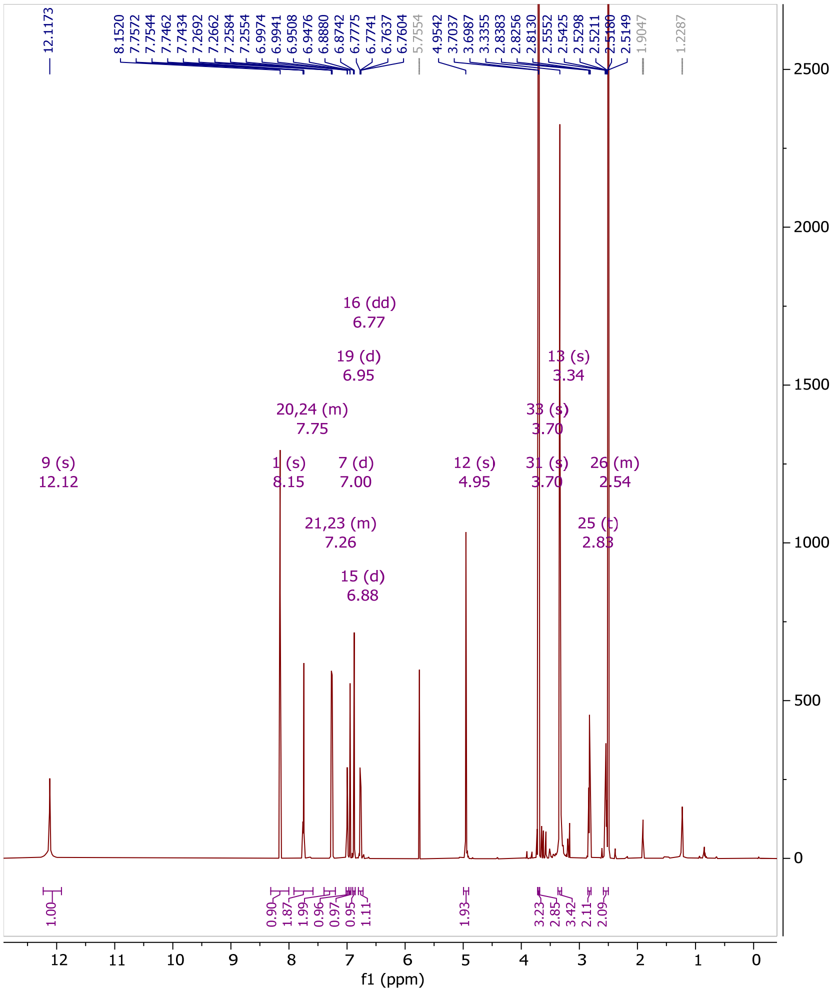

	Parameter	Value
1	Data File Name	C:/ Users/ Frithjof/ Desktop/ fab-04-33-fk/ 1/ fid
2	Title	fab-04-33-fk.1.fid
3	Comment	PROTON DMSO {C:\ Users\ nmrsu\ Documents} Frithjob 13
4	Origin	Bruker BioSpin GmbH
5	Owner	nmrsu
6	Site	
7	Instrument	spect
8	Author	
9	Solvent	DMSO
10	Temperature	298.0
11	Pulse Sequence	zg30
12	Experiment	1D
13	Probe	Z117768_0061 (CP TCI 600S3 H-C/ N-D-05 Z)
14	Number of Scans	32
15	Receiver Gain	11.5
16	Relaxation Delay	1.0000
17	Pulse Width	8.0000
18	Presaturation Frequency	
19	Acquisition Time	2.7263
20	Acquisition Date	2023-05-08T11:15:39
21	Modification Date	2023-05-08T11:15:39
22	Class	
23	Spectrometer Frequency	600.23
24	Spectral Width	12019.2
25	Lowest Frequency	-2308.0
26	Nucleus	1H
27	Acquired Size	32768
28	Spectral Size	131072
29	Digital Resolution	0.09

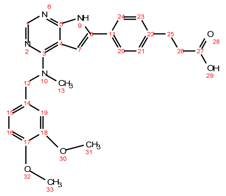

^{1}H NMR (600 MHz, DMSO-d_6) of L-2

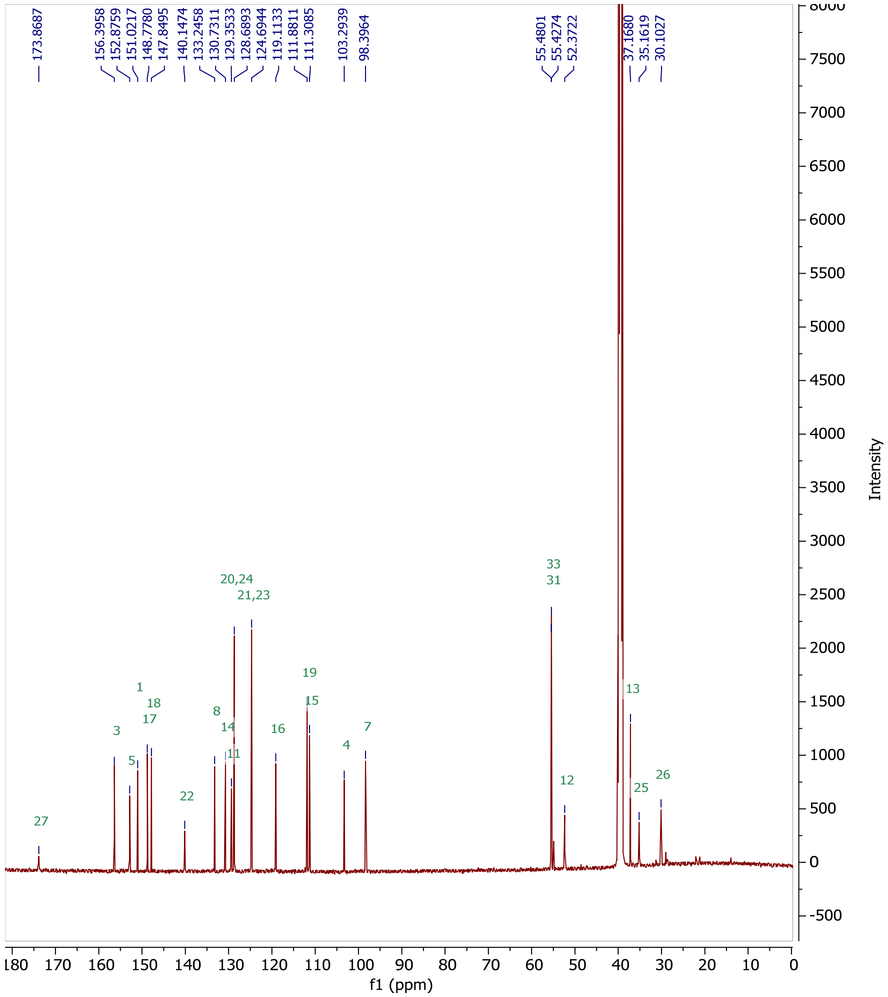

	Parameter	Value
1	Data File Name	C:/ Users/ Frithjof/ Desktop/ THP artikkel/ fab-04-33-fk/ 2/ fid
2	Title	fab-04-33-fk.2.fid
3	Comment	C13CPD_NTNU DMSO {C:\ Users\ nmrsu\ Documents} Frithjob 13
4	Origin	Bruker BioSpin GmbH
5	Owner	nmrsu
6	Site	
7	Instrument	spect
8	Author	
9	Solvent	DMSO
10	Temperature	298.0
11	Pulse Sequence	zgpg30
12	Experiment	1D
13	Probe	Z117768_0061 (CP TCI 600S3 H-C/ N-D-05 Z)
14	Number of Scans	5120
15	Receiver Gain	197.1
16	Relaxation Delay	2.0000
17	Pulse Width	11.4000
18	Presaturation Frequency	
19	Acquisition Time	0.9088
20	Acquisition Date	2023-05-08T15:30:13
21	Modification Date	2023-05-08T15:30:13
22	Class	
23	Spectrometer Frequency	150.94
24	Spectral Width	36057.7
25	Lowest Frequency	-3008.1
26	Nucleus	13C
27	Acquired Size	32768
28	Spectral Size	65536
29	Digital Resolution	0.55

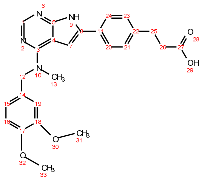

^{13}C NMR (150 MHz, DMSO-d_6) of L-2
