# Supplementary material for: Activation of multiple stress responses in Staphylococcus aureus substantially lowers the minimal inhibitory concentration when combining two novel antibiotic drug candidates
Source: Front Microbiol. 2023 Sep 25;14:1260120. doi: 10.3389/fmicb.2023.1260120 (PMC10564113; doi:10.3389/fmicb.2023.1260120)
Supplement: Supplementary file 1 [file Data_Sheet_1.zip › Table S3.PDF]

Supplementary Table 3: Metabolite abbreviations. TCA = tricarboxylic acid; PPP = pentose phosphate pathway

Class	Abbreviation	Metabolite
TCA cycle	Cit	Citric acid
	ICit	Isocitric acid
	aKG	α-Ketoglutaric acid
	Suc	Succinic acid
	Fum	Fumuric acid
	Mal	Malic acid
Glycolysis and PPP	G6P	Glucose 6-phosphate
	F6P	Fructose 6-phosphate
	F1,6BP	Fructose 1,6-bisphosphate
	2-/3PG	2-/3-Phosphoglyceric acid
	PEP	Phosphoenolpyruvic acid
	6PG	6-Phosphogluconic acid
	P5P	Pentose 5-phosphate
	S7P	Sedoheptulose 7-phosphate
Other sugar phosphates	M1P	Mannose 1-phosphate
	G1P	Glucose 1-phosphate
	F1P	Fructose 1-phosphate
	Gal6P	Galactose 6-phosphate
	GL3P	Glycerol 3-phosphate
	GA6P	Glucosamine 6-phosphate
	M6P	Mannose 6-phosphate
	MonoP	Monophosphate
	UDP-Glc/GalNAc	Uridine diphosphate-N-acetylglucosamine
	PRPP	Phosphoribosyl pyrophosphate
Nucleoside phosphates	AMP	Adenosine monophosphate
	ADP	Adenosine diphosphate
	ATP	Adenosine triphosphate
	GDP	Guanosine diphosphate
	GTP	Guanosine triphosphate
	CMP	Cytidine monophosphate
	CDP	Cytidine diphosphate
	CTP	Cytidine triphosphate
	UMP	Uridine monophosphate
	UDP	Uridine diphosphate
	UTP	Uridine triphosphate
Deoxynucleo-side phosphates	dATP	Deoxyadenosine triphosphate
	dGTP	Deoxyguanosine triphosphate
	dCTP	Deoxycytidine triphosphate
	dTTP	Deoxythymidine triphosphate
